# Paishi granule inhibits calcium oxalate-induced oxidative stress kidney injury after gut microbiota transformation: a multi-omics analysis combined in *vivo* and in *vitro* study

**DOI:** 10.1186/s13020-026-01439-4

**Published:** 2026-06-25

**Authors:** Bingqi Zhang, Xingxu Pan, Xiaolin Xie, Shiqi Fan, Tiantian Tan, Danqi Li, Jingsong Jin, Qiushi Cao

**Affiliations:** 1 Hubei Province Academy of Traditional Chinese Medicine, Hubei Key Laboratory of theory and application research of liver and kidney in Traditional Chinese Medicine, Affiliated Hospital of Hubei University of Chinese Medicine, Hubei Provincial Hospital of Traditional Chinese Medicine, Wuhan, 430061 China; 2 School of Basic Medical Sciences, Hubei Shizhen Laboratory, Hubei University of Chinese Medicine, Wuhan, 430061 China; 3 College of Traditional Chinese Medicine, Hubei Shizhen Laboratory, Hubei University of Chinese Medicine, Wuhan, 430061 China; 4 Xiangyang Central Hospital, Affiliated Hospital of Hubei University of Arts and Science, Xiangyang, 441021 China

**Keywords:** Paishi granule, Gut microbiota, Transcriptome sequencing, 16S rRNA, Targeted metabolomics, Cell culture

## Abstract

**Background:**

Renal stones are a common clinical problem with a high recurrence rate. In recent years, many Chinese herbs have been reported to effectively ameliorate calcium oxalate via different mechanisms. Gut microbiota plays an important role in the regulation of stone formation. In this study, we establish a novel molecular framework to illuminate that paishi granule (PSG) can treat calcium oxalate stone formation through gut microbiota transformation.

**Methods:**

The UPLC-MS/MS of serum-PSG was analyzed. A rat model of calcium oxalate kidney stones and a calcium oxalate monohydrate cell model (COM) were established. Blood samples were measured for Scr and BUN level. The histological staining and immunohistochemistry were used for observing pathological morphology of the kidneys and the colon. 16S rRNA sequencing, fecal metabonomics analysis, RNA transcriptomics sequencing, enzyme-linked immunosorbent assays, RT-qPCR, and western blotting were used to identify the signal transduction pathway of PSG in treating renal calculi to systematically explain the function of PSG after transformation by the gut microbiota.

**Results:**

PSG significantly alleviated renal injury induced by calcium oxalate stones, reduced BUN and Scr levels, and exerted antioxidant effects by enhancing T-SOD activity while decreasing LDH and MDA levels. Histological analysis of intestinal tissues demonstrated that PSG repaired intestinal barrier damage in EN rats. 16S rRNA sequencing revealed a decrease in the abundance of genera such as *Romboutsia, Clostridium*, and *Enterobacter*, and an increase in that of *Blautia, Acetatifactor,* and *Kineothrix* in EN group. SCFAs metabonomics analysis indicated significant reductions in acetate, propionate, and butyrate levels, which were improved after treatment with PSG, effectively ameliorating the disordered microbiota structure and metabolite profiles. The key differentially expressed genes, including *β-arrestin2, TGF-β1, and NOX4*, were identified using transcriptomics analysis, and enrichment analysis suggested the involvement of the MAPK signaling pathway. RT-qPCR and western blotting confirmed that PSG mitigated oxidative stress by suppressing the β-arrestin2/MAPK/NOX4 pathway. The renal protective effects of PSG were markedly attenuated in an antibiotic-induced model of gut-microbiota depletion. In *vitro* experiments further demonstrated that the PSG, after being transformed by the microbiota, could regulate the MAPK kinase cascade reaction through the mechanism mediated by *β-arrestin2*, inhibiting the process of *NOX4* expression.

**Conclusion:**

PSG exerts its anti-lithic effects through a novel multi-target regulatory mechanism involving the gut microbiota–metabolite–oxidative stress axis, thereby establishing a new paradigm for mechanistic research in traditional Chinese medicine.

**Supplementary Information:**

The online version contains supplementary material available at 10.1186/s13020-026-01439-4.

## Background

Kidney stones represent one of the most common urological disorders worldwide, with a steadily rising annual prevalence and a global incidence as high as 14.8% [1–3]. Among the various types of renal stones, calcium oxalate stones constitute up to 80% of cases, and their pathogenesis is closely linked to oxidative stress. Oxalate overload can activate NADPH oxidase in renal tubular epithelial cells, resulting in excessive mitochondrial reactive oxygen species accumulation while concurrently suppressing cellular antioxidant defense systems. This imbalance ultimately disrupts redox homeostasis and promotes the progression of nephrolithiasis [4, 5]. Sustained oxidative stress further exacerbates renal parenchymal injury and significantly increases the risk of end-stage renal disease [6]. Although advances in minimally invasive techniques—such as percutaneous nephrolithotomy and flexible ureteroscopy—have improved the management of renal stones, the 5-year recurrence rate remains as high as 50% after the first surgery [7]. This underscores the pressing need to identify novel therapeutic and preventive strategies that are both effective and associated with minimal side effects.

The gut microbiota, often referred to as the “second genome” of the human body, forms a complex interactive network with the nervous system, immune system, and endocrine system of the host through the gut–organ axis, and its metabolites play a pivotal role in disease regulation [8–10]. Recent studies have revealed that short-chain fatty acids (SCFAs), such as acetate, propionate, and butyrate, which are produced by the gut microbiota via fermentation of dietary fiber, can act as signaling molecules. They participate in the pathogenesis of kidney diseases via G protein–coupled receptor signaling or histone deacetylase–inhibition pathways [11–13]. For example, butyrate can alleviate adenine-induced chronic kidney disease fibrosis by suppressing NLRP3 inflammasome–mediated pyroptosis [14, 15]. A hyperglycemic environment disrupts the diversity of the gut microbiota in diabetic nephropathy, reduces SCFAs levels, and exacerbates lipid-metabolism disorders and insulin resistance [16]. Moreover, specific gut microbiota such as *Lactobacillus* can promote oxalate degradation by expressing formyl-CoA transferase, thereby reducing calcium oxalate deposition in the kidneys [17]. These findings highlight the crucial regulatory role of gut microbiota and their metabolites in calcium oxalate stone formation.

Traditional Chinese Medicine (TCM) has been widely applied in the prevention and treatment of urolithiasis, with certain herbal medicines and their active constituents demonstrating notable efficacy in inhibiting calcium oxalate stone formation [18, 19]. Compared with single-herb preparations, which often fail to provide comprehensive treatment addressing both symptoms and underlying causes, compound TCM formulas exhibit therapeutic advantages through their multi-pathway and multi-target mechanisms. Recent studies have shown that core herbs in Sanjin Paishi Decoction—*Lysimachiae Herba* (Jinqiancao), *Lygodii Spora* (Haijinsha), and *Galli Gigerii Endothelium Corneum* (Jineijin)—can effectively prevent calcium oxalate crystallization by modulating the gut microbiota [20]. Moreover, as most TCM compounds are absorbed in the gastrointestinal tract, they inevitably interact with the intestinal flora. The gut microbiota can convert poorly absorbable macromolecular or highly polar TCM components into bioavailable forms through processes such as glycoside hydrolysis [21]. Additionally, the diverse gut microbiome harbors specialized “metabolic enzyme systems” capable of modifying the structures of TCM constituents, facilitating the absorption of pharmacologically active compounds or generating metabolites with enhanced therapeutic potency [22]. These intricate interactions between TCM and the gut microbiota not only reveal novel therapeutic strategies but also provide a promising avenue for elucidating the pharmacological mechanisms of TCM. The present study focuses on Paishi Granule (PSG), a clinically optimized formula consisting of 11 medicinal herbs: *Lysimachiae Herba, Lygodii Spora, Galli Gigerii Endothelium Corneum, Vaccariae Semen, Talcum, Ostreae Concha, Malvae Semen, Paeoniae Radix Alba, Glycyrrhizae Radix, Pyrrosiae Folium,* and *Toosendan Fructus*. Although PSG shows significant clinical efficacy in treating renal calculi [23], its precise mechanism of action in preventing and treating stone formation remains unclear.

In this study, we deduced that PSG can ameliorate the formation of calcium oxalate stones by the transformation of intestinal flora and regulating intestinal metabolites. By establishing a rat model of calcium oxalate stones, we found that the PSG significantly inhibited renal oxidative stress in a gut microbiota-dependent manner. Furthermore, an antibiotic-induced microbiota-depletion model confirmed that the therapeutic effect of PSG was markedly attenuated, indicating that the gut microbiota is essential for its anti-urolithiasis efficacy.

## Materials and methods

### Preparation of the qualified Chinese herbal compound PSG

PSG is an agreed prescription in the hospital consisting of 30 g of *Lysimachiae Herba*, 15 g of *Lygodium*, 15 g of *Endothecium Gigeriae Galli*, 15 g of *Vaccariae Semen*, 15 g of *Talcum*, 10 g of Oyster, 15 g of *Malva Seed*, 15 g of *Radix Paeoniae Alba*, 5 g of *Licorice*, 20 g of *Pyrrosiae Folium*, and 15 g of *Fructus Toosendan*. All Chinese herbal decoction pieces were provided by Hubei Tianji Pharmaceutical Co., Ltd. (Wuhan, China). First, 10 volumes of pure water were used to soak the TCM pieces for 2 h. After complete soaking, the herbs were boiled at high heat for 40 min. The residue and the extract were filtered and separated. The extract was decocted again and concentrated to yield a suspension containing the crude preparation of PSG at a mass concentration of 1.53 g/mL. The sample was refrigerated at –20 °C until further use. The main components of PSG are shown in Table 1.

**Table 1:** Composition of PSG

Chinese name	Latin name	Weight **G**	Part used
JinQianCao	*Lysimachiae Herba*	30 g	Herb
JiNeiJin	*Galli Gigeriae Endothelium*	15 g	Chicken gizzard membrane​
HaiJinSha	*Lygodii Spora*	15 g	Seed
HuaShi	*Talcum*	15 g	Mineral
ShengMuLi	*Ostrea gigas*	10 g	Mussel
DongKuiZi	*Malva verticillata* L	15 g	Seed
BaiShao	*Paeoniae Radix Alba*	15 g	Root
GanCao	*Licorice*	5 g	Root
ShiWei	*Pyrrosiae Folium*	20 g	Tuber
ChuanLianZi	*Toosendan Fructus*	15 g	Fruit
WangBuLiuXing	*Vaccariae Semen*	15 g	Seed

### Animal experiments and experimental design

Six-week-old specific-pathogen-free (SPF) grade male Sprague–Dawley (SD) rats weighing 200 ± 20 g were provided by Liaoning Changsheng Biotechnology Co., Ltd. (license No. SCXK [Liao] 2020–0001). The study protocol was approved by the Ethics Committee of Hubei University of Traditional Chinese Medicine (batch No.: HUCMS 7768272). The rats were housed in a Specific Pathogen-Free (SPF) facility at the Animal Experimental Center of Hubei University of Traditional Chinese Medicine. The rats were maintained under controlled environmental conditions (20–24℃, 40%–70% relative humidity) with a 12-h light/dark cycle. All animals had ad libitum access to ^60^Co-irradiated feed and sterile ultrapure water.

In the initial phase of the study, 24 rats were randomly allocated into four groups (n = 6/group). The control group (Ctrl) received a standard laboratory diet. To establish a calcium oxalate urolithiasis model, the remaining 18 rats were administered 1% ethylene glycol and 2% ammonium chloride via oral gavage as lithogenic agents[24]. Following model establishment, these 18 rats received daily intragastric treatments as follows: the EN group received 2 mL of ammonium chloride solution; the PSG group received 2 mL of PSG extract (equivalent to 1.53 g/mL of crude herbal material); and the SHC group received 2 mL of 5% potassium sodium hydrogen citrate solution.

In the second phase of the experiment, 30 Sprague–Dawley (SD) rats were randomly assigned to five groups (n = 6/group): Ctrl, EN, Ab, AbEN, and AbENP. Based on the modeling protocol established in the first phase, the antibiotic-treated groups received an antibiotic cocktail (0.5 g/L ampicillin, 0.25 g/L vancomycin, 0.5 g/L neomycin, and 0.5 g/L metronidazole) dissolved in their sterile drinking water [25]. After a 28-day induction period, serum, urine, and kidney tissue samples were collected from all rats.

### Biochemical testing of serum and urine samples

The levels of all biochemical indicators were tested following the manufacturers’ instructions in the respective kits. Scr, BUN, and LDH levels were determined using a fully automated biochemical analyzer from Wuhan Servicebio Biotechnology Co., Ltd. (Wuhan, China). Enzyme-linked immunosorbent assay kits for kidney injury molecule (KIM-1) and colorimetry kits for total superoxide dismutase (T-SOD) (WST-1 method) and malondialdehyde (MDA) (TBA method) were provided by Elabscience Biotechnology Co., Ltd. (Wuhan, China). A kit for the determination of oxalic acid content was procured from Beijing Solarbio Technology Co., Ltd. (Beijing, China).

### Hematoxylin and eosin (H&E), Von Kossan, and Alcian blue staining

Kidney tissues and intestinal tissue were fixed using paraformaldehyde, embedded in paraffin, and sectioned. H&E staining and Von Kossan staining were used to stain kidney sections, and Alcian blue was used to stain intestinal sections.

### Immunohistochemistry

First, 4-µm-thick sections of colon tissues were prepared following standard immunohistochemical procedures. The tissue sections of the corresponding groups were incubated with the primary antibodies ZO-1 (1:200, ab276131, Abcam), Occludin (1:200, ab216327, Abcam), and SLC26A6 (1:200, ab217269, Abcam) overnight at 4 °C. The tissues were then incubated with anti-rabbit secondary antibody (horseradish peroxidase HRP-labeled secondary antibody immunoglobulin g [IgG] multimer, 1:2000, ab205718, Abcam) at room temperature for 2 h. After each step, the sections were washed with phosphate-buffered saline (PBS), developed with DAB, counterstained with hematoxylin, and sealed with neutral resin. ImageJ software was used to quantitatively analyze the expression regions representing positivity.

### 16S rRNA sequencing

Fecal samples from rat cecum were collected and stored in sterile Eppendorf tubes in a refrigerator at –80℃. After extracting fecal genomic DNA, the genomic DNA of the drawer was analyzed using 1% agarose gel electrophoresis. 16 s RNA gene amplification was performed according to the designated V3–V4 region, and fusion primers with misaligned bases were synthesized. Magnetic beads were used to screen the self-linked fragments without linkers, and polymerase chain reaction (PCR) amplification was used to enrich the library template to obtain single-stranded DNA fragments. The DNA fragments and base primers were complemented to start sequencing, in which modified DNA polymerase and 4 types of fluorescence-labeled dNTP were added. The fluorescence intensity signals collected each time were counted to obtain the sequence on the template DNA fragment. The top 10 species in each group were screened using abundance as an index. Alpha diversity analysis was described using the Chao1 and Shannon indices, and beta diversity analysis was described using PCoA. Linear discriminant analysis–Effect size (Lefse) analysis was used to compare species that were significantly different between groups.

### Fecal targeted metabolomics

The sample of 100 mg was added with the corresponding volume of 80% aqueous methanol solution, mixed, centrifuged at 12,000 rpm at 4℃ for 10 min, and 50 μL of supernatant was taken, 150μL of derivatization reagent was added, derivatized at 40 ℃ for 40 min, and the derived sample was diluted with 80% aqueous methanol solution to the corresponding multiple. 95 μL of the supernatant was added to 5 μL of 80% aqueous methanol solution of the mixed internal standard, mixed, and subjected to LC–MS analysis. An ultra-high performance liquid chromatography coupled to tandem mass spectrometry (UHPLC-MS/MS) system (Vanquish™ Flex UHPLC-TSQ Altis™, Thermo Scientific Corp., Germany) was used to quantitate SCFAs level. Separation was performed on a Waters ACQUITY UPLC BEH C18 column (2.1 × 100 mm, 1.7 μm) which was maintained at 40 °C. The mass spectrometer was operated in negative multiple reaction mode (MRM) mode. Parameters were as follows: IonSpray Voltage (−4500 V), Sheath Gas (35psi), Ion Source Temp (550 °C), Auxiliary Gas (50psi), Collision Gas (55psi).

### Transcriptome sequencing

Transcriptomics analysis was performed on the Ctrl, EN, and PSG groups. Total RNA was extracted from kidney tissue samples, and the concentration and purity of the extracted RNA were determined using a NanoDrop2000 spectrophotometer (NanoDrop, Wilmington, Delaware, USA). RNA integrity was determined using agarose gel electrophoresis, and RIN values were obtained using an Agilent 2100 bioanalyzer (Agilent Technologies, Santa Clara, CA, USA). Under the condition of single-library establishment, the total RNA amount should be ≥ 1 µg and the concentration should be ≥ 35 ng/µL. After the library was established, second-generation high-throughput sequencing platform was used for sequencing, and the sequencing results were stored in FASTQ file format. HISAT2 software was used to align the filtered sequencing sequences of each sample with the reference genome to locate and determine the relevant gene information. Lastly, BLAST software was used to align all genes with Swiss-Prot, GO and KEGG databases to enrich gene-related functional information.Transcriptomics accession number: PRJNA1400648; (http://www.ncbi.nlm.nih.gov/bioproject/1400648).

### Cell culture and transfection

Rat proximal tubular epithelial cells (NRK-52E) were purchased from Promega Corporation (Catalog No.: CL-0174, Wuhan, China). The cells were cultured in a medium containing 5% fetal bovine serum and 1% double antibody (penicillin–streptomycin solution) (Dulbecco’s Modified Eagle Medium) in an incubator flushed with 5% CO_2_ at 37 °C. To establish a calcium oxalate cell model (COM), oxalic acid (Ox, Sigma-Aldrich, 379,735-5 g) was prepared in complete medium at concentrations of 250, 500, 750, and 1000 μmol/L. NRK-52E cells were cultured, and cell proliferation activity was determined. The oxalate concentration of COM was determined to be 1 mM Ox. Lastly, the COM cell model was treated with drug-containing serum (Serum-PSG and Serum-AbPSG), and the therapeutic effects were analyzed. To knock down *β-arrestin2* (*ARRB2*) expression, *β-arrestin2* mimics and control mimics (Genepharma, China) were synthesized using RNA oligo and introduced into NRK-52E cells according to the manufacturer’s instructions.

### Cell viability assay and determination of biochemical indices

To determine the activity of NRK-52E cells in different groups, the treated cells were analyzed using a 10% cell counting kit-8 assay (CCK-8, MCE, HY-K0301) and incubated at 37 °C for 2 h. The optical density (OD) was measured at 450 nm using spectrophotometry. The antioxidant activity of cells in different groups was determined using a malondialdehyde (MDA) kit (Nanjing Chengjian Biotechnology Research Institute, A003-1–1) and a total-superoxide dismutase (T-SOD) kit (Nanjing Chengjian Biotechnology Research Institute, A001-3–1).

### RT-qPCR analysis of mRNA

HyperScriptTM III RT SuperMix for RT-qPCR with gDNA remover reverse-transcription synthesis kit IV was used. Total RNA was extracted from rat kidneys, and its concentration and purity were determined. Reverse transcription was performed using a SPARK script II RT Plus kit (Sparkjade, China). RT-qPCR was performed using a LightCycler 480 instrument II (Roche, Germany), and the Ct values of the target genes were corrected using β-actin. Seven primer sequences were designed and are listed in Table 2.

**Table 2:** Primers for reverse transcription–quantitative polymerase chain reaction

Gene	Forward primer sequence	Reverse primer sequence
*β-arrestin2*	AGTATGCCGACATTTGCCTCTTCAG	GAGACACCACCAGCTTCACCTTG
*TGF-β1*	CCACGCTCTTCTGTCTACTGAACTTC	GGTATGAAATGGCAAATCGGCTGAC
*NOX4*	GCCCTCTTACTGTGTCCTACTGAAAC	AACCACTGGAATGATTGGATGTCTCTG
*ERK1/2*	CGGTACTATGAGAAGGAGCACAATGAG	GCCACCAGAGACACAACAAATACAAC
*JNK*	TGGATTTGGAGGAGCGAACTAAGAATG	TCATCTACAGCAGCCCAGAGGTC
*p38MAPK*	AACCTCGCTGTGAATGAAGACTGTG	CTTGGGCTGCTGTGATCCTCTTATC
*β-Actin*	GTCACCAACTGGGACGATA	GAGGCATACAGGGACAACA

### Western blotting

Rat kidneys were homogenized using a grinder in RIPA lysis buffer (Servicebio, China) containing 1% phenylmethylsulfonyl fluoride, 1% phosphatase inhibitor A, 1% phosphatase inhibitor B, and 2% 50 × cocktail (Servicebio, China). Protein concentrations were determined using an enhanced bicinchoninic acid protein assay kit (Servicebio, China). The proteins were separated using sodium dodecyl sulfate–polyacrylamide gel electrophoresis and transferred to polyvinylidene fluoride membranes (Servicebio, China). After incubation with 5% skimmed milk powder in Tris-buffered saline containing 0.1% Tween-20 for 30 min, the membranes were incubated with the primary antibody *β-actin* (E-AB-48018, 1:1000, Elabscience); *NOX4* (E-AB-70215, 1:1000, Elabscience); *β-arrestin2* (10,171–1-AP, 1:1000, Proteintech); *TGF-β1* (21,898–1-AP, 1:1000, Proteintech); *P38MAPK* (14,064–1-AP, 1:2000, Proteintech); *ERK1/2* (E-AB-31374, 1:500, Elabscience); *JNK* (E-AB-60070, 1:500, Elabscience); *phospho-ERK* (E-AB-70310, 1:500, Elabscience); *phospho-P38MAPK* (AF4001, 1:500, Affinity); *phospho-JNK* (AF3318, 1:500, Affinity) at 4 °C. The sections were then incubated with HRP-conjugated goat anti-rabbit IgG (1:5000) for 30 min. Protein signals were observed using an enhanced chemiluminescence protein detection kit (Biosharp, China). Target signals of proteins were normalized to β-actin and quantified using ImageJ software.

### Statistical analysis methods

Data were analyzed using GraphPad Prism 10.1.2. Continuous data are presented as mean ± standard deviation (x ± s). Comparisons between two groups were performed using the independent samples t-test, and comparisons across multiple groups were conducted using one-way analysis of variance (ANOVA). For data that did not follow a normal distribution, the Kruskal–Wallis test was used for multiple comparisons. A *P*-value < 0.05 was considered statistically significant.

## Results

### Analysis of UPLC-MS/MS of serum-PSG

Utilizing a serum pharmacochemistry strategy, this study systematically characterized the serum containing drug components and metabolic profiles of PSG and AbPSG (prepared under normal and flora-depleted conditions, respectively) to elucidate their absorptive behavior and metabolic disparities. Firstly, using blank serum as a control, UPLC-MS/MS technology was employed to identify 170 components in Serum-PSG and 167 components in Serum-AbPSG, preliminarily clarifying the material basis of the two drug-containing serum samples. Further, through a one-way comparison of the original solution and drug-containing serum, combined with differential analysis, a hierarchical comparison screening mode of "serum—original solution—flora status" was ultimately adopted to select four differentially entering components: emodin, scutellarin, taurodeoxycholic acid, and isoschaftoside (Fig. S1A). It was speculated that these components, as key active ingredients of traditional Chinese medicine, as the result of microbial metabolism, enter the circulatory system, and their key substances have been clearly identified.

In the analysis using ultra-high performance liquid chromatography-tandem mass spectrometry (UPLC-MS/MS), based on the total ion current chromatograms of negative and positive ion modes, multi-dimensional screening was conducted using retention time (RT), mass-to-charge ratio (m/z), and peak area response values. The top 10 compounds with the highest response intensity were determined, including PSG characteristic components that were bioconverted by the intestinal flora (Fig. S1B, C). Through high-resolution mass spectrometry ion fragment analysis, the chromatographic behavior and molecular characteristics of the target components were further confirmed: emodin (RT = 7.52 min, m/z = 269.0452), scutellarin (RT = 4.79 min, m/z = 285.0401), taurodeoxycholic acid (RT = 4.36 min, m/z = 464.2823), and isoschaftoside (RT = 4.46 min, m/z = 565.1541) (Fig. S1D). This provided precise chemical basis for subsequent mechanism studies.

Molecular docking simulations demonstrated that all four components exhibited strong binding affinities to the key target protein NADPH oxidase 4 (NOX4), yielding binding free energies (ΔG) of −7.62, −7.58, −9.20, and −8.13 kcal/mol, respectively (Fig. S1E). Notably, these four components displayed particularly robust binding capacities, suggesting they may modulate oxidative stress by inhibiting NOX4 activity. Furthermore, network pharmacology analysis revealed that the targets of these bioactive components were significantly enriched in KEGG pathways, including 'Mineral absorption' and the 'MAPK signaling pathway' (Fig. S2A,B,D,E). Gene Ontology (GO) enrichment analysis indicated that the core targets were primarily involved in biological processes such as the inflammatory response, MAPK cascade, and response to reactive oxygen species (Fig. S2C,F). Collectively, these findings provide systematic insights into the potential pharmacological mechanisms underlying the therapeutic effects of the medicated serum.

### PSG protects from calcium oxalate–induced oxidative stress and kidney damage

**Fig. 1:**
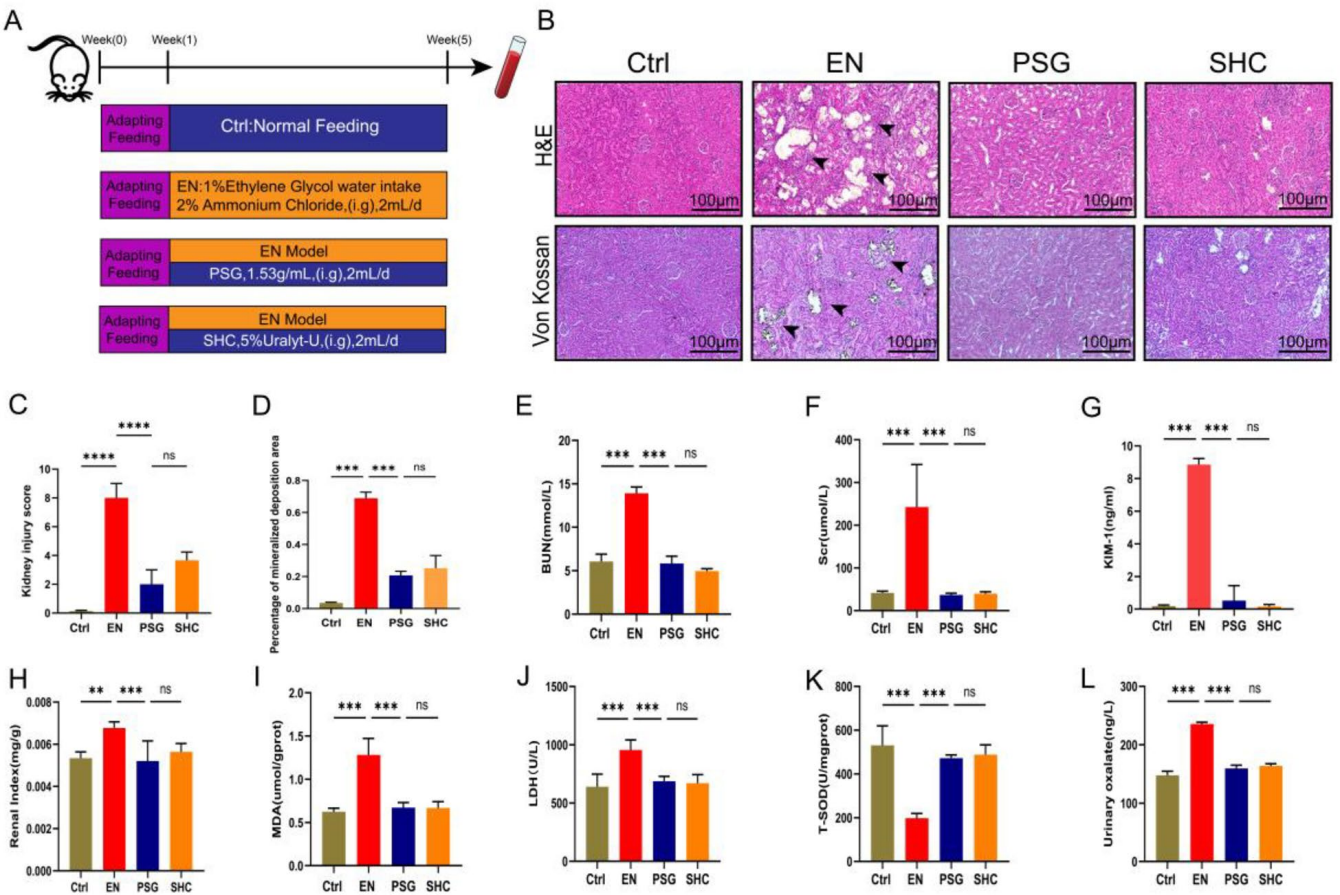
Establishment of a rat model of calcium oxalate stones and the protective effects of PSG against calcium oxalate-induced oxidative stress and renal injury. **A** Experimental arrangement and grouping of model animals. **B** H&E staining and Von Kossan staining of rat kidneys (scale 100 µm, 200 ×). **C** Kidney injury score. **D** Mineralized deposition score. **E** Blood urea nitrogen (BUN). **F** Serum creatinine (Scr). **G** Kidney injury molecule (KIM)−1. **H** Kidney index. **I** Malondialdehyde (MDA). **J** Lactate dehydrogenase (LDH). **K** Total superoxide dismutase (T-SOD). **L** Urinary oxalate. Data are presented as mean ± standard deviation (n = 6). *P < 0.05, **P < 0.01, ***P < 0.001 compared with EN, ^*ns*^*P* > 0.05 compared with PSG

A rat model of calcium oxalate kidney stone was established by feeding 6-week-old male SD rats for 1 week, followed by providing 1% ethylene glycol in drinking water and 2% ammonium chloride solution by gavage. After 4 weeks of continuous intervention, H&E staining of the renal tissues of rats exhibited different degrees of tubular expansion and the deposition of pale-yellow calcium oxalate crystals (black arrows). Von Kossan staining revealed the deposition of dark-brown calcium oxalate crystals (black arrows) (Fig. 1A-B). The indices related to oxidative stress and renal function injury were determined to evaluate the protective effect of PSG in the kidneys of rats with calcium oxalate. PSG could alleviate kidney damage and reduce calcium oxalate crystal deposition in the renal tubules of rats compared with EN group (*P* < *0.05*) (Fig. 1C-D). Analysis of the other biochemical indicators revealed varying increases in the levels of Scr, BUN, KIM-1, the kidney index, and other indicators (Fig. 1E-H). Calcium oxalate kidney stones could promote the excretion of urinary oxalic acid (Fig. 1L) and simultaneously induce an increase in the oxidative stress markers MDA and LDH, and decrease the levels of the antioxidant T-SOD (Fig. 1I-K). This trend could be ameliorated by administering PSG and potassium sodium hydrogen citrate granules.

### PSG alleviates calcium oxalate–induced intestinal barrier damage and dysfunction

**Fig. 2:**
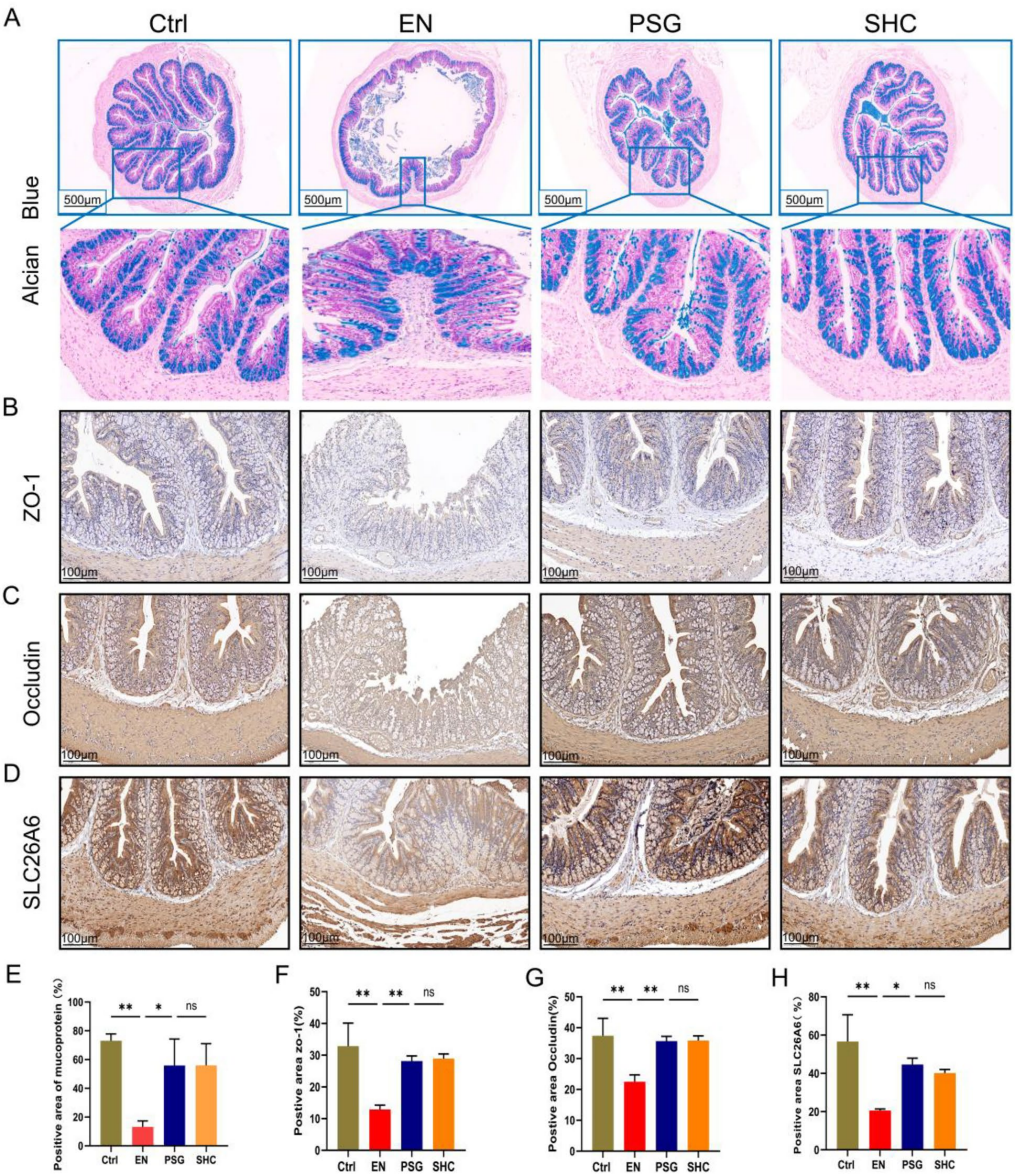
Pathological changes in the colons of model rats with calcium oxalate kidney stones and the changes after PSG intervention. **A** Alcian blue staining of colon tissues (scale 500 µm, 100 ×). **B** Immunohistochemical staining for ZO-1 in the colon. **C** Immunohistochemical staining for Occludin in the colon. **D** Immunohistochemical staining for SLC26A6 in the colon (above immunohistochemistry image specification: scale 100 µm, 200 ×). **E** Quantitative analysis of intestinal mucin. **F** Quantitative analysis of intestinal ZO-1. **G** Quantitative analysis of intestinal Occludin. **H** Quantitative analysis of intestinal SLC26A6. Data are presented as mean ± standard deviation (n = 6). *P < 0.05, **P < 0.01, compared with EN, ^*ns*^*P* > 0.05 compared with PSG

Structural changes in the colon tissues of rats with calcium oxalate stones were analyzed using histological staining to determine the protective effect of PSG on maintaining the integrity of the intestinal barrier. Alcian blue staining showed edema in the colon mucosa of rats with EN group, a thin and discontinuous muscle layer, and a decrease in the content of glycoproteins and mucins in the colon (Fig. 2A). However, PSG intervention inhibited the calcium oxalate–induced decrease in glycoproteins and mucins (*P* < *0.05*) (Fig. 2E). Findings from immunohistochemistry revealed that the ZO-1 and Occludin were downregulated in the colons of mice in the EN group (Fig. 2B, C), whereas PSG could reverse the downregulated expression of these tight junction proteins in the colons of rats bearing calcium oxalate (Fig. 2F, G). Moreover, the expression of SLC26A6 in the colon was determined, and the trend was consistent with the above results (Fig. 2D, H).

### PSG adjusts the abundance of the gut microbiota in rats with calcium oxalate calculi

**Fig. 3:**
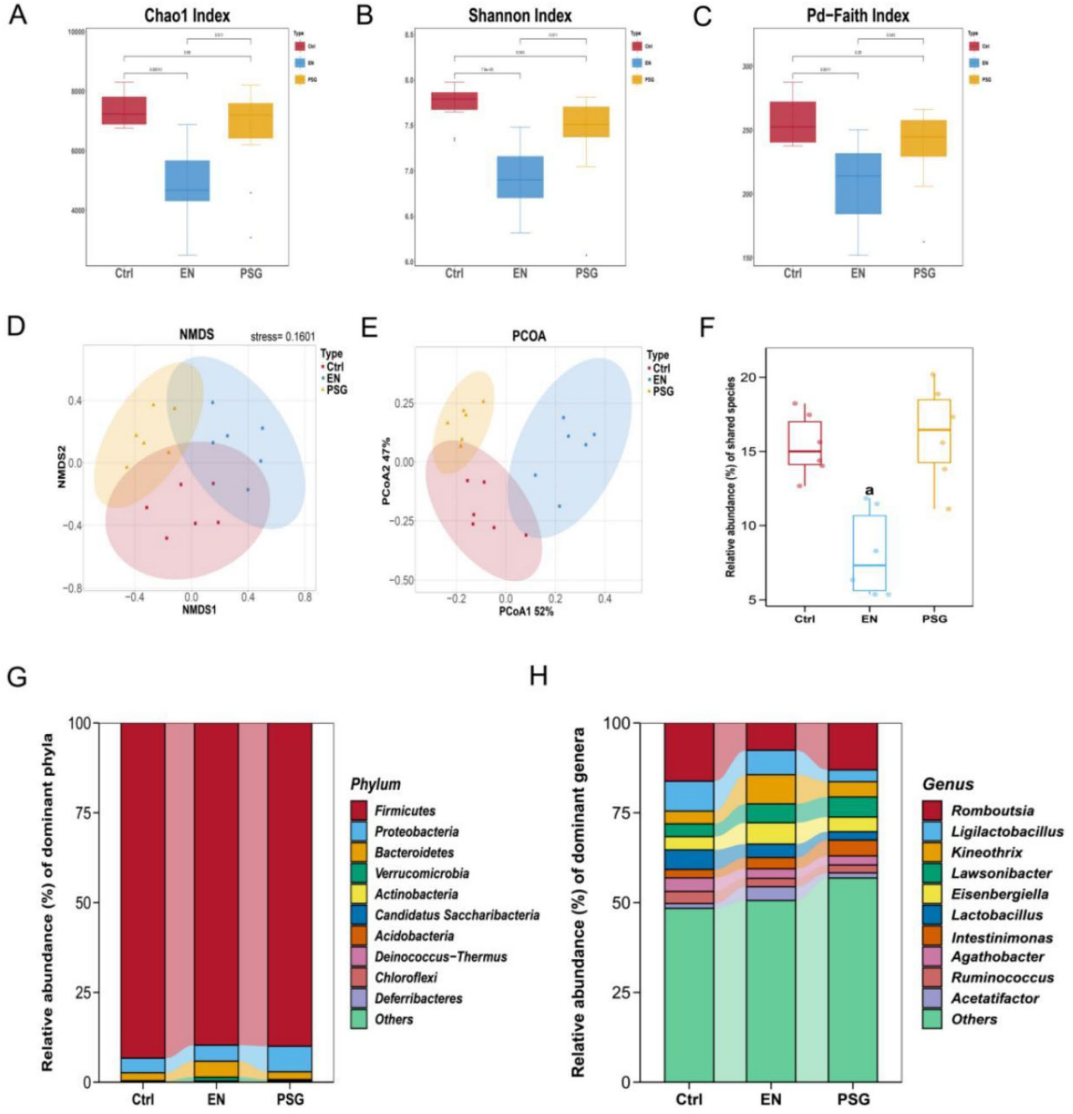
Effect of calcium oxalate kidney stones on the gut microbiota in rats and the effects after PSG intervention. Alpha diversity analysis included **A** Chao1 index, **B** Shannon index, and **C** Pd-Faith index. Beta diversity analysis included **D** NMDS and **E** PCoA. **F** Comparison of the richness of shared species. **G** Comparative bar graph of the relative richness at the phylum level. **H** Comparison bar chart of the relative richness of genus. Data indicate significant differences: ^*a*^*P* < 0.05 compared with the Ctrl

Given the therapeutic efficacy of PSG against calcium oxalate, 16S rRNA gene sequencing was utilized to evaluate its modulatory effects on the gut microbiota. Alpha diversity analysis revealed that microbial richness, overall species diversity, and phylogenetic diversity—indicated by the Chao1, Shannon, and Faith's PD indices, respectively—were significantly decreased in the EN group compared to the Ctrl group (Fig. 3A–C). Furthermore, Beta diversity analyses, including Non-metric Multidimensional Scaling (NMDS) and Principal Coordinate Analysis (PCoA), demonstrated distinct structural clustering among the three groups, indicating that calcium oxalate stone induction significantly altered the overall architecture of the gut microbiota (Fig. 3D, E). The relative abundance of the shared species was compared in the three groups, and a similar trend was observed as that obtained for the Alpha diversity analysis (Fig. 3F). Taxonomic profiling of relative abundances further delineated these microecological shifts. At the phylum level, the EN group exhibited a pronounced depletion of *Firmicutes* and an enrichment of *Bacteroidetes* relative to the Ctrl group, a dysbiotic trend that was effectively reversed following PSG intervention (Fig. 3G). Similarly, at the genus level, PSG treatment successfully restored the stone-induced depletion of *Romboutsia* and the aberrant accumulation of *Kineothrix* and *Acetifactor* (Fig. 3H). Finally, Linear discriminant analysis Effect Size (Lefse) was employed to robustly identify specific differential taxa. Lefse identified *Clostridium-saudiense*, *Romboutsia-ilealis*, and *Enterobacter -roggenkampii* as the keystone species enriched in the PSG-treated rats, whereas *Blautia-*sp.*001304935*, *Acetifactor-*sp.*900066565*,and*Kineothrix -alysoides* emerged as primary biomarkers for the EN group (Fig. S3).

**Fig. 4:**
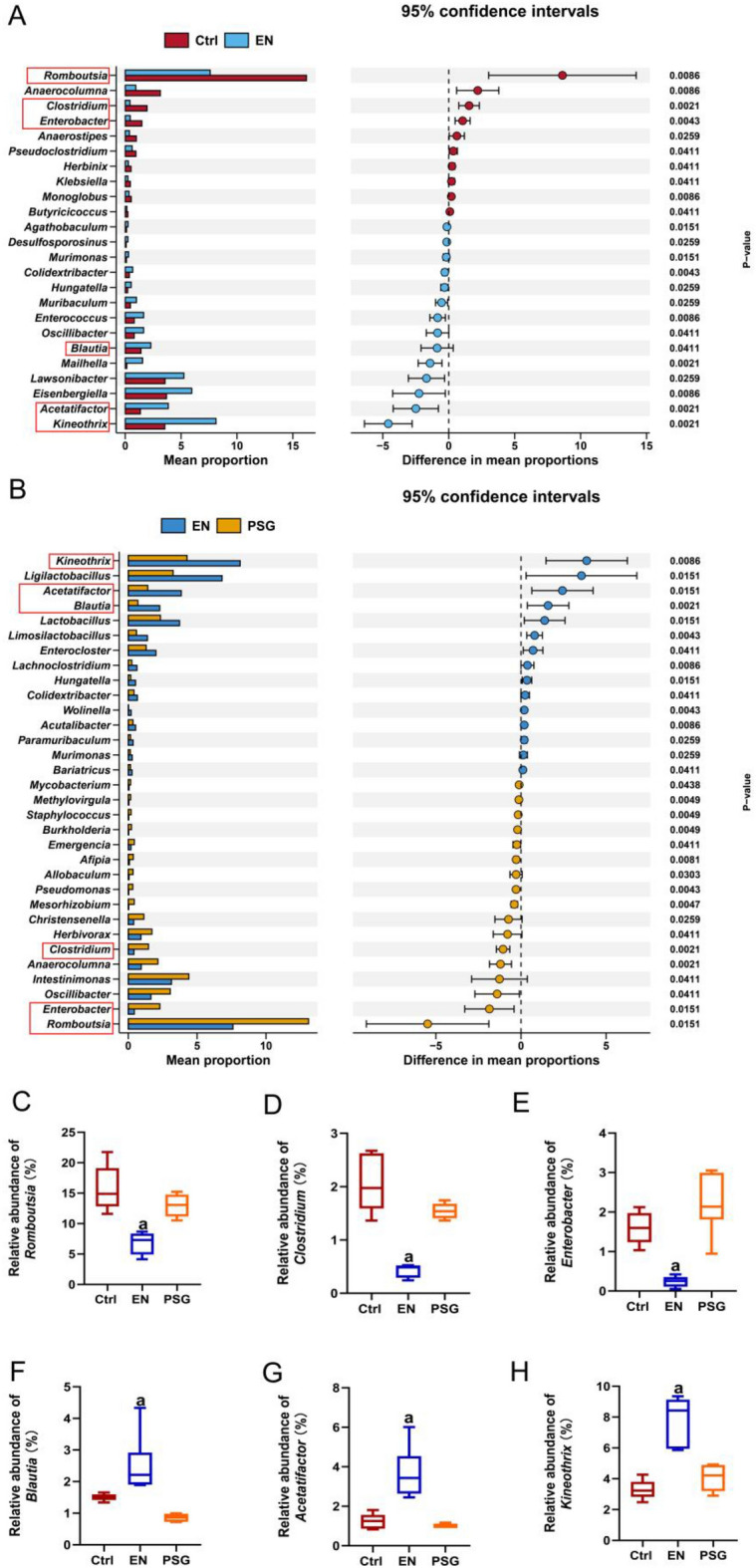
Differential analysis of the gut microbiota using 16S rRNA data. **A** Wilcoxon analysis of the Ctrl vs EN groups. **B** Wilcoxon analysis of the EN vs PSG groups. (**C-H**) Quantitative analysis of the relative abundance of differential bacteria common to the Ctrl, EN, and PSG groups. Data are presented as mean ± standard deviation (n = 6). ^*a*^*P* < 0.05 compared with the Ctrl

The Wilcoxon rank-sum test was used to analyze the Ctrl vs EN and the EN vs PSG groups to further clarify the differences in bacterial genera among the three groups. The nonparametric test to compare two independent samples was used, and the findings revealed that the abundances of *Romboutsia, Clostridium, Enterobacter, Blautia, Acetatifactor*, and *Kineothrix* were consistent with those determined using Lefse and that they came from the same bacterial genera (Fig. 4A, B). Subsequent quantitative analysis of the above bacterial species revealed the ability of PSG in regulating the gut microbiota (Fig. 4C–H).

### PSG adjusts SCFAs in the fecal metabolites of rats with calcium oxalate calculi

**Fig. 5:**
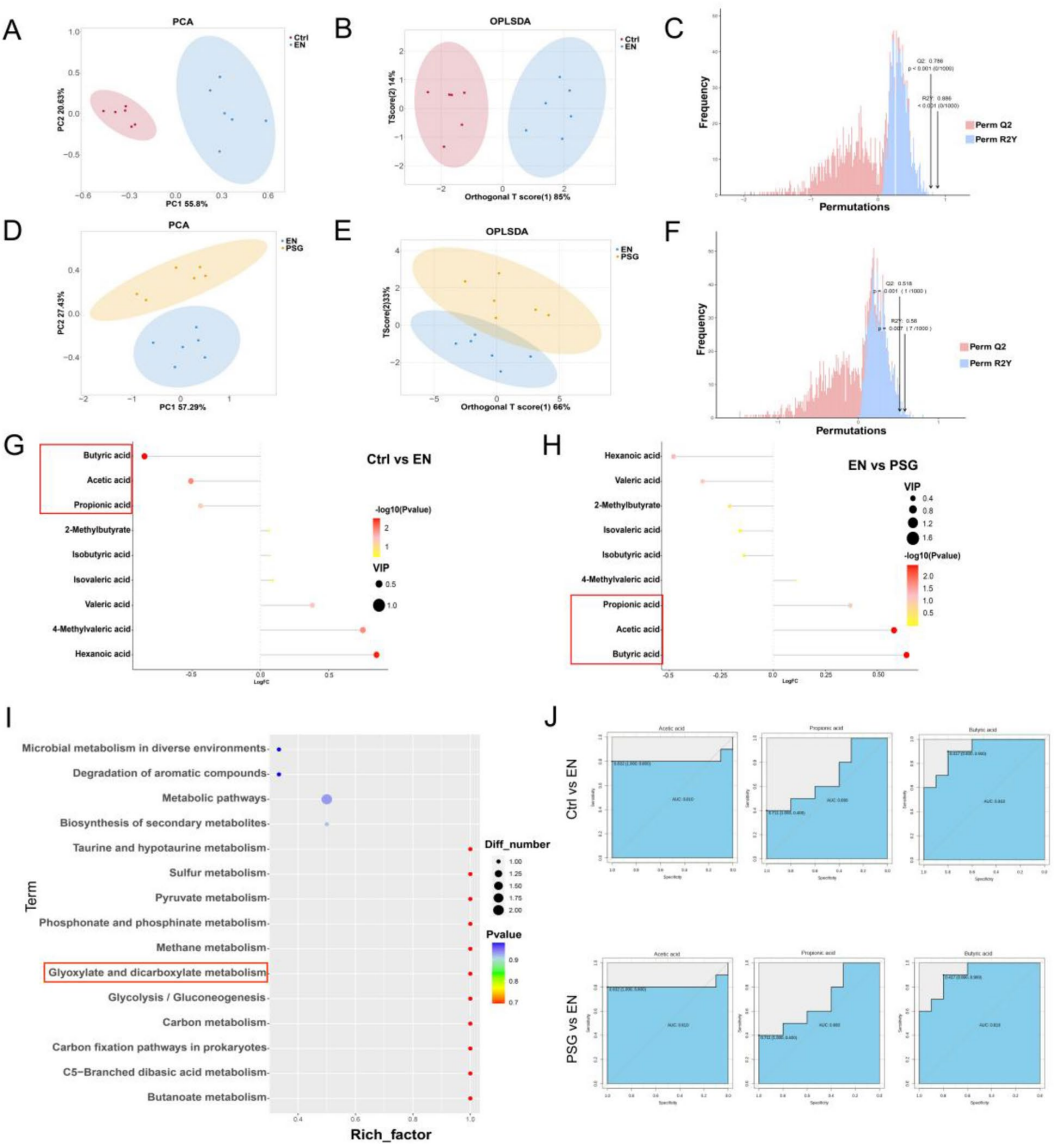
Targeted metabolomics of SCFAs in fecal samples. **A** PCA plot of Ctrl vs EN. **B** OPLS-DA plot of Ctrl vs EN. **C** Substitution test chart for OPLS-DA (Ctrl vs EN). **D** PCA plot of EN vs PSG. **E** OPLS-DA plot of EN vs PSG. **F** Substitution test chart for OPLS-DA(EN vs PSG). **G** Ctrl vs EN metabolite SCFAs differential matchstick plot. **H** EN vs PSG metabolite SCFAs differential matchstick plot. **I** KEGG enrichment profile of SCFAs metabolites. **J** SCFAs metabolite survival curves (ROC)

Next, changes in the composition and richness of microbial SCFAs metabolites were determined, and targeted metabolomics was used to analyze SCFAs. The Ctrl vs EN groups were compared, followed by the comparison of the EN vs PSG groups. Quantitative results of SCFAs metabolites were analyzed using PCA. The samples in the two groups showed some discrimination, and the trend of intergroup separation in the experimental model was observed using PCA (Fig. 5A, D). Then OPLS-DA is used to filter signals unrelated to the classification among different groups. Accordingly, the OPLS-DA model was used to compare metabolites that were significantly different between the two groups (Fig. 5B, E), indicating changes in the metabolic spectrum of bacteria in the EN group. The validity of the OPLS-DA model was verified using the permutation test, where R^2^ represents the interpretation rate of the established model and Q^2^ represents the prediction ability of the model. Theoretically, the closer the R^2^ and Q^2^ values are to 1, the better is the model (Fig. 5C, F). The R^2^ and Q^2^ values to be > 0.5, indicating that the model stability determined using OPLSDA for the two groups was excellent and without any overfitting.

Therefore, the differential metabolites in SCFAs were quantitatively calculated and compared. Taking log2 as the base of logarithmic transformation, a lollipop plot showing the top 10 differential metabolites is presented (Fig. 5G, H). The plot shows the fold change after logarithmic transformation on the abscissa, the size of the point represents the variable importance in projection value, and the color represents the *P*-value of the *t*-test of the metabolite. Acetic acid, propionic acid, and butyric acid ranked higher in the common differential metabolites of the Ctrl group, EN group, and PSG group. However, enrichment analysis of the metabolic pathways of the differential metabolites revealed that SCFAs participated in the metabolism of glyoxylic acid and dicarboxylic acid (Fig. 5I, J). Glyoxylic acid was determined to be the intermediate product of the metabolism of ethylene glycol in rats in our study. PSG could reverse the changes in the intestinal metabolites caused due to calcium oxalate stones in rats and affect oxalic acid metabolism.

### PSG ameliorates calcium oxalate stones via the inhibition of theβ-arrestin2/MAPK/NOX4 signaling axis.

**Fig. 6:**
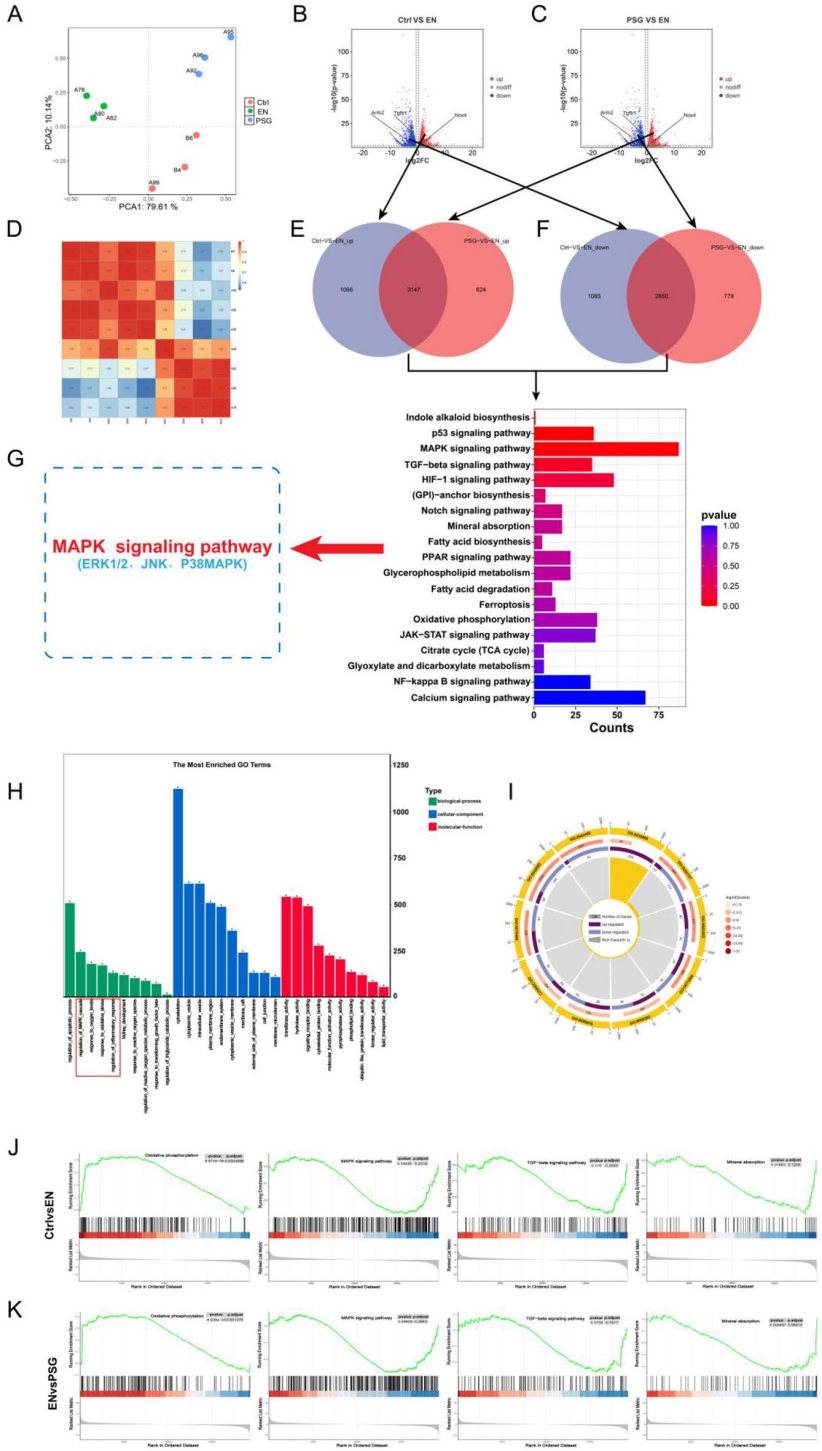
Transcriptomic sequencing analysis of kidney tissue samples. **A** Principal component analysis. **B** Ctrl vs EN difference analysis volcano map. **C** EN vs PSG difference analysis volcano map. **D** Heatmap of within-group sample correlations. **E** Ctrl vs EN Venn diagram. **F** EN vs PSG Venn diagram. **G** KEGG enrichment analysis of intersection genes (*P*-value < 0.05). **H** Intersection gene GO enrichment analysis bar graph. **I** Circle diagram of GO enrichment analysis of intersection genes. **J** Ctrl vs EN GSEA enrichment analysis. **K** EN vs PSG GSEA enrichment analysis

To elucidate the underlying pharmacological mechanisms of PSG against calcium oxalate nephrolithiasis, transcriptomic sequencing of kidney tissues was conducted. Principal component analysis (PCA) and Pearson correlation algorithms demonstrated distinct inter-group stratification and robust intra-group reproducibility among the Ctrl, EN, and PSG groups (Fig. 6A, D). Differential expression analysis, visualized via volcano plots and Venn diagrams, identified a consensus set of differentially expressed genes (DEGs) common to the CtrlvsEN and PSGvsEN comparisons. Notably, crucial target genes including *Arrb2* (encoding *β-arrestin2*), *NOX4*, and *TGF-β1* were significantly modulated (Fig. 6B, C). Firstly, compile the sets of commonly up-regulated and down-regulated genes from the CtrlvsEN and PSGvsEN comparisons. Functional enrichment analysis is then performed on the combined set of these differential genes. (Fig. 6E, F). Based on the KEGG pathway enrichment results, considering both the count of enriched genes and the statistical significance (lower P-value indicates higher significance), the MAPK signaling pathway was selected for further study(Fig. 6G). GO enrichment analysis of differential genes revealed that the biological processes that could be enriched included regulation of MAPK cascades, response to oxidative stress, and response to reactive oxygen species (Fig. 6H,I). Gene Set Enrichment Analysis (GSEA) was conducted to directly use gene expression quantity ranking, using the log2FC of each difference group as the score of the background gene set to analyze the enrichment of specific gene sets, and finally controlling the *P*-value as < 0.05 and the FDR as < 0.05 as a significantly enriched gene set (Fig. 6J, K). Based on the results from the enrichment analysis, it could be speculated that activation of the MAPK signaling pathway and the occurrence of oxidative stress reactions contribute to the pathogenesis of calcium oxalate kidney stones.

To experimentally validate these bioinformatics predictions, the mRNA and protein expression levels of key components within this axis—specifically *β-arrestin2*, *NOX4*, *TGF-β1*, *ERK1/2*, *p38 MAPK*, and *JNK*—were quantified in *vivo* utilizing RT-qPCR and Western blotting, respectively. The resulting experimental data corroborated the transcriptomic findings, strictly confirming that PSG intervention effectively suppresses the activation of the β-arrestin2/MAPK/NOX4 signaling cascade in the calcium oxalate kidney stone model (Fig. S4).

### Correlation analysis was performed to predict the associations between metabolites and the microbiota, as well as between metabolites and biomarker

**Fig.7:**
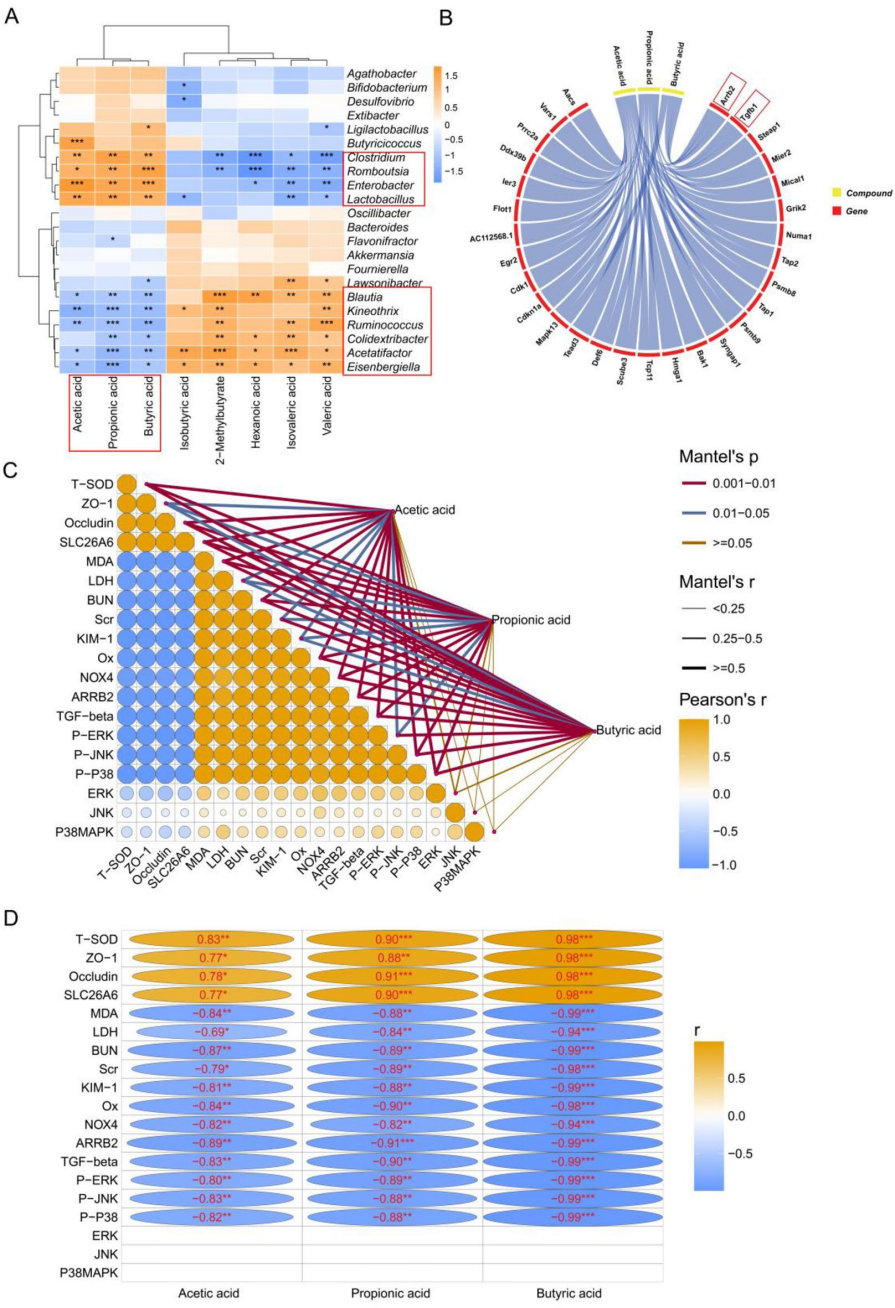
Correlation analysis of gut microbiota and metabolites, and correlation analysis of metabolites and target molecules. **A** Spearman correlation analysis of metabolites and gut microbiota. **B** Circle map of associations between SCFAs metabolites and transcriptome differences in genes. **C** Combination diagram of SCFAs and target molecules. **D** Pearson coefficient plot of correlation combination plot. n = 6 *P < 0.05, **P < 0.01 and **|r|> 0.5

Differentially expressed SCFAs metabolites and gut microbiota were preselected for Spearman correlation analysis. Acetic acid, propionic acid, and butyric acid in metabolite SCFAs were positively correlated with *Romboutsia, Clostridium,* and *Enterobacter*, and negatively correlated with *Blautia, Acetatifactor*, and *Kineothrix* (*P* < *0.05, P* < *0.01*) (Fig. 7A). These findings suggest that SCFAs production is regulated by a type of beneficial bacteria and a type of harmful bacteria in the colon. Next, correlation analysis was performed based on the differentially expressed genes and differentially expressed metabolites obtained from each group in the transcriptome and metabolome. The positive and negative absolute values of the correlation coefficients that were ≥ 0.9 were selected. The acetic acid, propionic acid, and butyric acid were correlated with *β-arrestin2* and *TGF-β1* in the differential genes of the transcriptome (Fig. 7B). Pearson correlation analysis was used to verify the correlation between SCFAs and the key markers. Based on the positive and negative absolute values of the Pearson correlation coefficient r approaching 1, the results showed that Acetic acid, propionic acid, and butyric acid were positively correlated with T-SOD, ZO-1, Occludin, and SLC26A6, whereas *NOX4, P-ERK, P-P38, P-JNK*, MDA, Ox, *β-arrestin2, TGF-β1*, BUN, Scr, and LDH were negatively correlated with the metabolites (r|> 0.5) (Fig. 7C, D). The findings of this predictive analysis suggested that SCFAs could mediate oxidative stress–induced renal damage due to calcium oxalate stones.

### PSG failed to alleviate the injury of calcium oxalate stones in rats with depleted gut microbiota

**Fig. 8:**
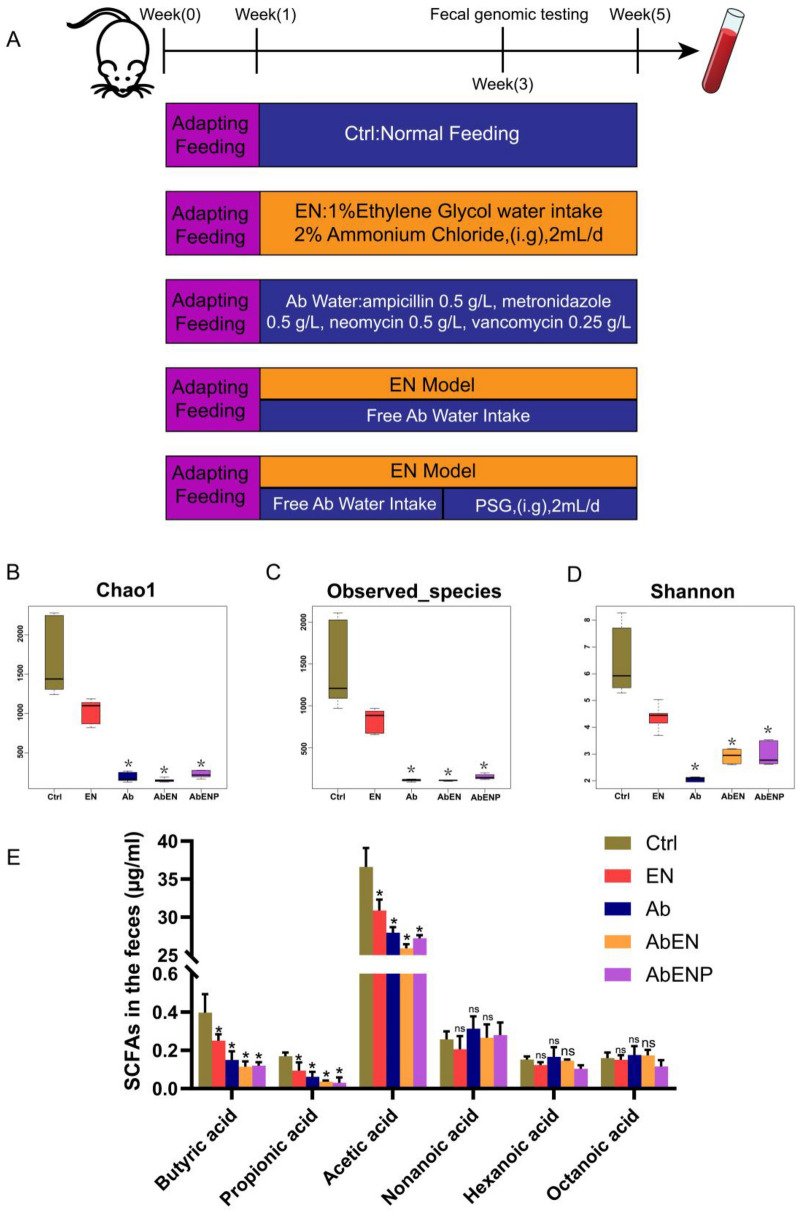
Effect of PSG on rats with calcium oxalate stones in the gut microbiota depletion model. **A** Experimental arrangement of the calcium oxalate model under antibiotic intervention. **B** Chao1 index. **C** Observed species index. **D** Shannon index. **E** Quantitative analysis chart of SCFAs. The number of samples in each group was n = 6, and the significance is expressed as *P < 0.05, ^*ns*^*P* > 0.05 compared with the Ctrl group

**Fig. 9:**
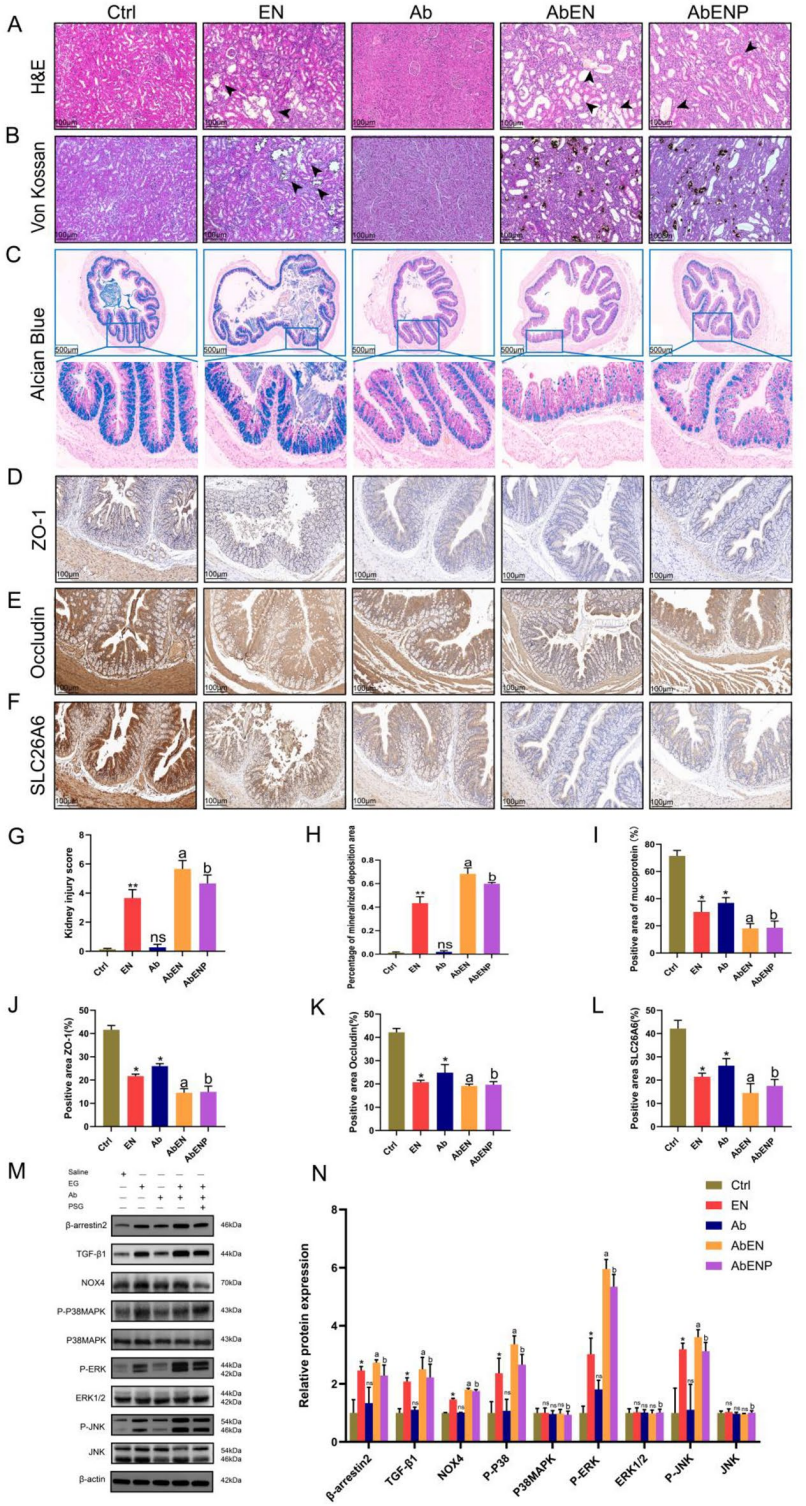
The prerequisite for the kidney-protective effect of PSG on calcium oxalate is a complete gut microbiota. **A** H&E staining of the kidney tissues of rats. **B** Von Kossan staining of the kidney tissues of rats. **C** Alcian blue staining of the colon tissues of rats. **D** Immunohistochemical staining for ZO-1 in the colon tissues of rats. **E** Immunohistochemical staining for Occludin in the colon tissues of rats. **F** Immunohistochemical staining for SLC26A6 in the colon tissues of rats. **G** Kidney injury score. **H** Mineralized deposition score. **I** Quantitative analysis of Alcian blue staining. **J** Immunohistochemical quantitative analysis of ZO-1. **K** Immunohistochemistry quantitative analysis of Occludin. **L** Immunohistochemical quantitative analysis of SLC26A6. **M** Western blotting to determine protein expression. **N** Quantitative analysis of protein expression. Data are presented as mean ± standard deviation (n = 6). *P < 0.05, ^*ns*^*P* > 0.05 compared with the Ctrl, ^*a*^*P* < 0.05 compared with Ab, ^*b*^*P* > 0.05 compared with AbEN

Based on the above findings, the role of PSG in treating calcium oxalate kidney stones and exerting its anti-oxidative stress effect was further explored by focusing on the transformation of the gut microbiota. Antibiotic intervention was undertaken in this study on the basis of the calcium oxalate kidney stone model. Rats were treated with an antibiotic mixture (Ab) for 4 weeks to eliminate gut microbiota to determine the therapeutic effect of PSG on calcium oxalate formation (Fig. 8A). Fecal samples were subjected to 16S rRNA sequencing and targeted metabolism testing to confirm successful establishment of the bacterial-depletion model. Analysis revealed a significant reduction in both alpha diversity and SCFAs levels (acetic, propionic and butyric acid) in the Ab group relative to the Ctrl group. (Fig. 8B–E). Pathological changes in the kidney tissues of rats in the bacterial-depletion model were analyzed using HE and Von Kossan staining. Kidney tissues from rats in the Ab group were comparable to those in the Ctrl group and showed no signs of abnormality. (Fig. 9A, B). Moreover, the kidney injury score and the stone deposition score were not significantly different between the simple Ab and Ctrl groups (^*ns*^*P* > 0.05) (Fig. 9G, H). The intestinal structure in Ab-treated mice was observed using Alcian blue staining. Mucin expression and the number of intestinal villi decreased, and the size of the intestinal villi appeared shrunken compared with that Ctrl group (Fig. 9C, I). These findings indicated that antibiotic intervention could deplete the intestinal flora without damaging the kidneys. However, H&E and Von Kossan staining of the pathological sections of the kidneys of rats with AbEN group revealed that the pathological damage did not improve significantly after PSG intervention (Fig. 9A, B, G, H). Furthermore, PSG could not reverse the trend of the decrease in the expression of SLC26A6 and the ZO-1 and Occludin in the intestinal barrier in rats with AbEN group (Fig. 9D, E, F, J, K, L). Overall, our findings suggest that the ability of PSG to treat renal calculi and ameliorate intestinal metabolism is dependent on the integrity of the gut microecosystem.

### By transformation of the gut microbiota, PSG inhibits COM–induced oxidative stress by regulating the β-arrestin2/MAPK/NOX4 signaling pathway

**Fig. 10:**
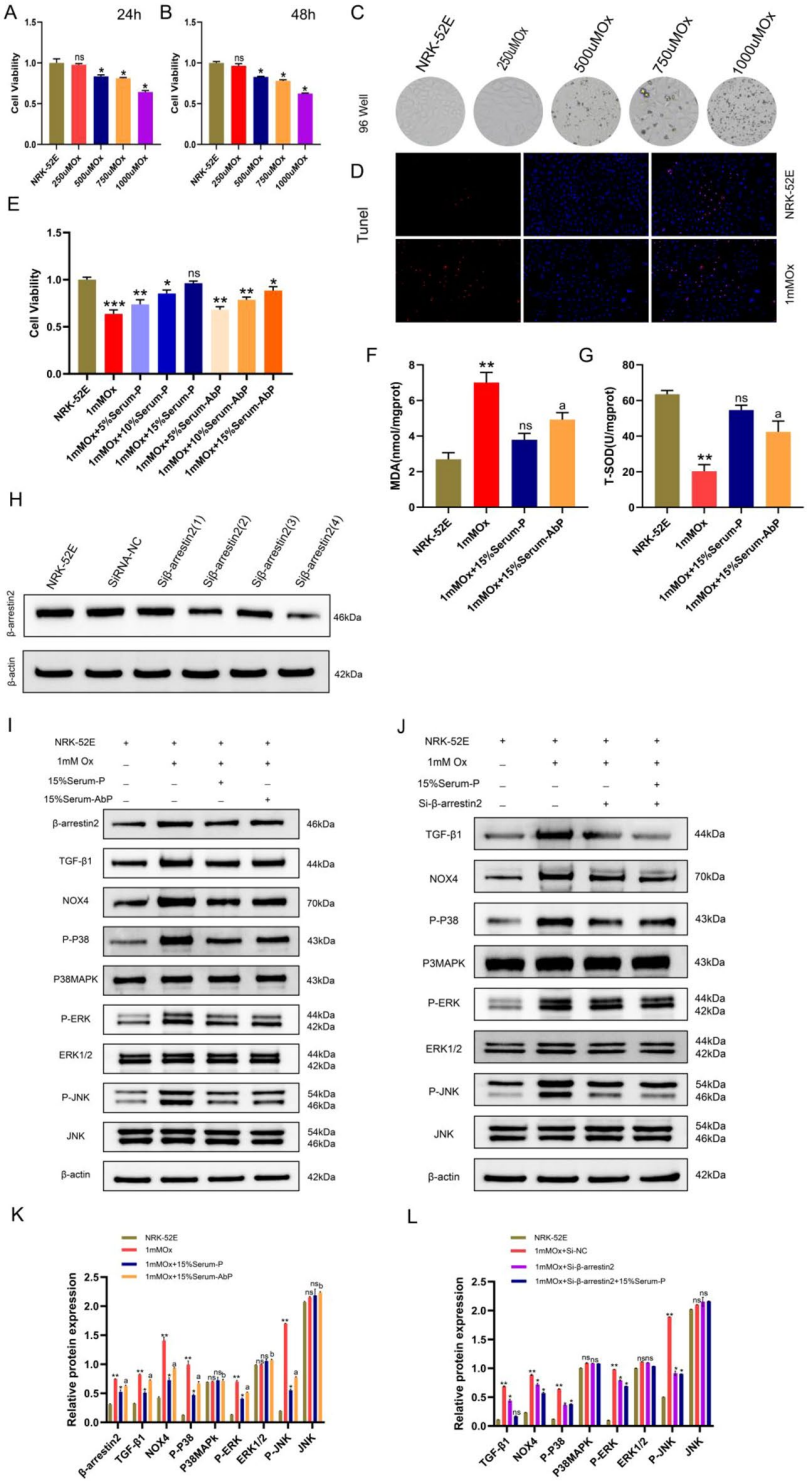
After transformation by the gut microbiota, Serum-PSG could inhibit (COM)-induced oxidative stress by regulating the β-arrestin2/MAPK/NOX4 signaling pathway. **A** Cell viability of NRK-52E cells after treatment with different concentrations of oxalic acid (250, 500, 750, and 1000 μmol/L) for 24 h. **B** Cell viability after treatment with different concentrations of oxalic acid for 48 h. **C** Morphology of cells stimulated by COM and observation using an inverted microscope (200 ×). **D** Determination of cell apoptosis using TUNEL staining. **E** Effects of Serum-P and Serum-AbP on the viability of NRK-52E in COM model. **F** Changes in T-SOD activity in the COM cell model. **G** Changes in MDA levels in the COM cell model. **H** Determination of the efficiency of siRNA knockdown of β-arrestin2 using western blotting. **I** Effect of PSG after transformation by the gut microbiota on COM-induced damage. **J** Serum-PSG regulates oxidative stress in COM-treated cells through the β-arrestin2–mediated MAPK signaling pathway. **K** Quantitative analysis of related proteins expression in the Serum-PSG treatment group. **L** Quantitative analysis of related proteins expression in the si-β-arrestin2 treatment group. Data are presented as mean ± standard deviation (n = 6). *P < 0.05, ^*ns*^*P* > 0.05 compared with the NRK-52E, ^*a*^*P* < 0.05 compared with 1mMOx + 15%Serum-P, ^*b*^*P* > 0.05 compared with 1mMOx + 15%Serum-P

Based on differentially expressed genes (including *β-arrestin2, NOX4*, and *TGF-β1*) and the MAPK signaling pathway screened using transcriptome sequencing, The primary aim of this study was to investigate the effects of PSG on the expression of associated target molecules in the renal tissue of calcium oxalate stone-forming rats subjected to microbiota depletion. The protein expression of the β-arrestin2/MAPK/NOX4 signaling pathway in the EN and AbEN group was significantly increased compared with that in the Ctrl group (*P < 0.05, ^*a*^*P* < 0.05); however, PSG failed to significantly reverse the abnormal protein expression in the kidneys of rats with kidney stones when the abundance of microbiota was depleted (Fig. 9M, N). To establish a calcium oxalate monohydrate (COM) model in NRK-52E cells, 1 mM oxalic acid was used. Cell viability was then assessed following exposure using the CCK-8 assay (Fig. 10A, B). To verify the validity of the model, NRK-52E cells were observed using microscopy after stimulation with COM. The growth rate showed a decrease, cell morphology was disturbed, and deposition of calcium oxalate crystals was noted (Fig. 10C). Further, TUNEL staining revealed that in the COM model (NRK-52E cells treated with 1 mM oxalate), apoptotic cells were labeled with red fluorescence. These cells displayed characteristic morphological features of apoptosis, including nuclear shrinkage, fragmentation, and the formation of apoptotic bodies (Fig. 10D). Accordingly, serum concentrations (Serum-P and Serum-AbP) that can promote cell proliferation were screened, and the serum of PSG was found to effectively reverse COM-induced oxidative stress only after transformation by the intestinal flora (Fig. 10E–G). Furthermore, Western blotting was used to confirm that Serum-P could inhibit the upregulation of COM-induced β-arrestin2/MAPK/NOX4 signaling molecular protein more effectively than Serum-AbP. After the *β-arrestin2* gene was knocked down, the findings demonstrate that PSG effectively inhibits NOX4 protein expression by modulating the MAPK cascade through a β-arrestin2-mediated mechanism. (Fig. 10H–L).

**Fig. 11:**
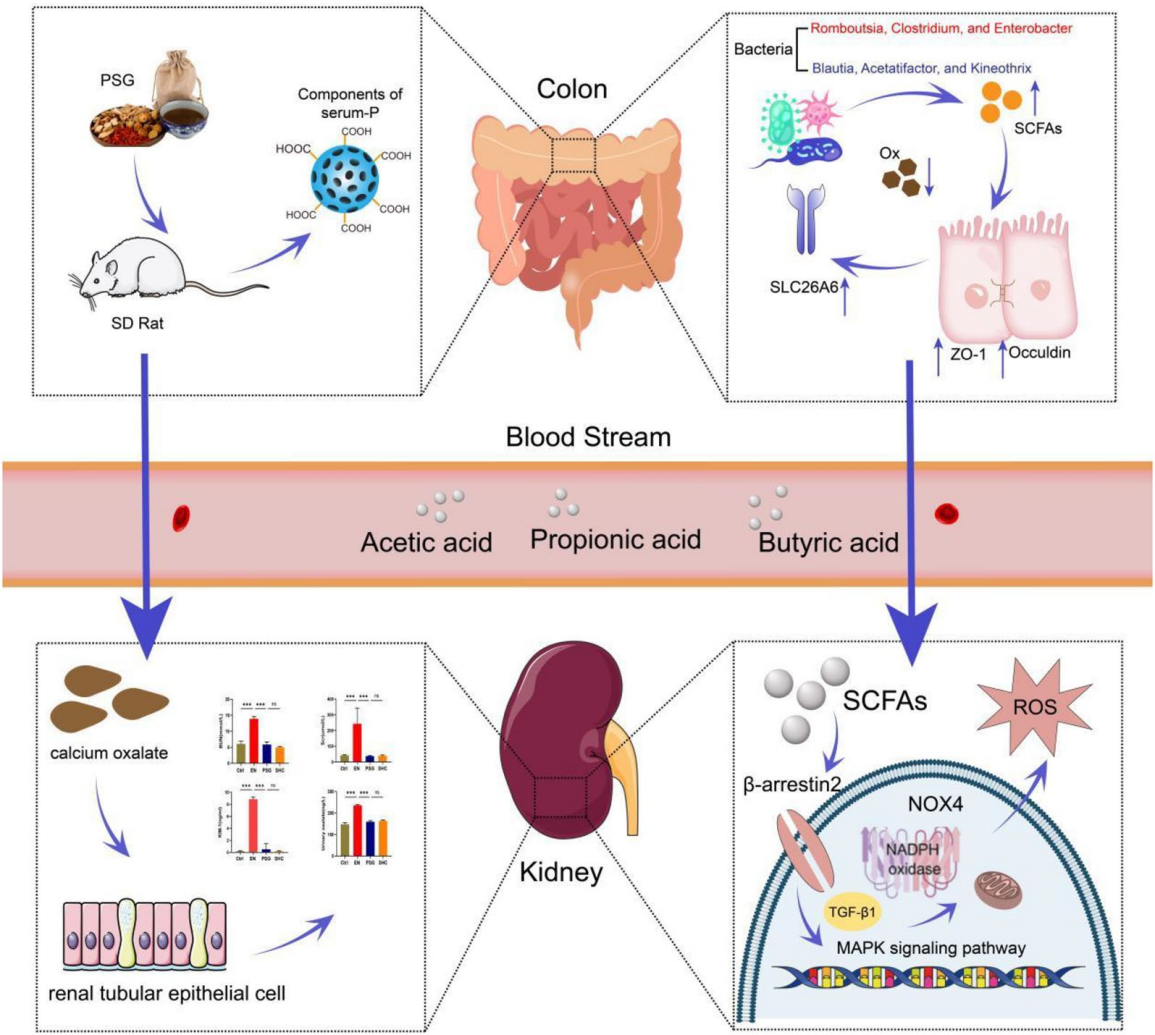
Molecular mechanism of PSG in regulating the gut microbiota and SCFAs in inhibiting calcium oxalate–induced oxidative stress

This study demonstrates that PSG alleviates calcium oxalate-induced oxidative renal injury by acting on the "microbiota-metabolite-oxidative stress" axis, specifically through restoring gut microbiota composition, increasing SCFAs levels, upregulating SLC26A6 expression, and inhibiting the β-arrestin2/MAPK/NOX4 signaling pathway (Fig. 11).

## Discussion

This study systematically investigated the preventive and therapeutic effects of PSG on renal injury in a calcium oxalate animal model, and elucidated the mechanism by which it regulates the gut microbiota–metabolite–oxidative stress axis. The main findings are as follows: (1) PSG significantly alleviated calcium oxalate-induced renal tissue damage and suppressed oxidative stress responses. (2) PSG ameliorated the reduction in expression of Occludin, ZO1, and SLC26A6 induced by calcium oxalate, thereby restoring intestinal barrier integrity and promoting enteric oxalate metabolism. (3) PSG effectively ameliorated the gut microbiota dysbiosis induced by calcium oxalate and restored the levels of microbiota-derived metabolites, specifically SCFAs. (4) In a microbiota-depletion model, the therapeutic effect of PSG on calcium oxalate stones was shown to depend on an intact gut microbial ecosystem. In conclusion, these results collectively support the hypothesis that PSG prevents and treats kidney stones via the gut–kidney axis through a multi-target, multi-dimensional regulatory mechanism.

The gut microbiota, a complex microbial community colonizing the human gastrointestinal tract, participates in key host physiological processes such as defense against exogenous infections, regulation of nutrient metabolism, and modulation of aging [26]. A systematic review has shown that patients with kidney stones exhibit significant alterations in gut microbiota composition [27]. In the present study, a rat model of calcium oxalate kidney stones demonstrated a decreased abundance of genera such as *Romboutsia, Clostridium,* and *Enterobacter*, alongside an increased abundance of genera including *Blautia, Acetatifactor,* and *Kineothrix*. Previous studies have reported that a higher abundance of *Romboutsia* contributes to enhanced SCFAs metabolism and alleviation of intestinal inflammation [28]. Another study indicated that supplementation with *Clostridium*, a high butyrate-producing genus, accelerated the restoration of intestinal flora homeostasis in subjects experiencing post-colonoscopy abdominal pain and diarrhea [29]. Furthermore, some evidence suggests that the genus *Acetatifactor* associated with liver and kidney injury induced by euphorbiaceae plants [30]. Based on these findings, we speculate that the host may be under the synergistic influence of two functionally distinct bacterial groups: one comprising "beneficial bacteria" that inhibit disease progression, and the other consisting of "harmful bacteria" that promote disease onset. This hypothesis is strongly supported by recent work from Liping Zhao’s team, which highlights that shifts in the core microbiota represent a key event in disease pathogenesis [31]. In this study, PSG was found to reverse the core microbiota dysbiosis induced by calcium oxalate kidney stones, further underscoring the important role of gut microbiota homeostasis in disease prevention and treatment.

Furthermore, the maintenance of intestinal barrier homeostasis mainly depends on a variety of key regulatory mechanisms: Intestinal mucin and tight junction proteins (such as ZO-1 and Occludin) respectively constitute the mucus physical barrier and the paracellular tight junction barrier that prevent the penetration of endotoxins and inflammatory factors [32, 33]. In addition, the oxalic acid transporter SLC26A6 also plays a key role in the intestinal oxalic acid metabolism process. This study found that under oxalate loading conditions, the expressions of intestinal tight junction proteins (ZO-1, Occludin) and SLC26A6 were significantly downregulated. PSG intervention can effectively improved the above changes, restore intestinal barrier function and enhance intestinal oxalic acid metabolism. These results suggest that PSG may play a role in the prevention and treatment of calcium oxalate stones by repairing the structure and function of the intestinal barrier and regulating the expression of SLC26A6 proteins, thereby restoring the overall metabolic balance of the intestinal microecology.

Based on the above-mentioned changes in the intestinal flora, fecal targeted metabolomics analysis further revealed that calcium oxalate load disrupted the normal metabolism of SCFAs. Short-chain fatty acids are mainly composed of acetic acid, propionic acid and butyric acid, accounting for more than 95% of the total. They are important metabolic products produced by the fermentation of dietary fiber by intestinal flora [34, 35]. The results of this study show that the levels of acetic acid, propionic acid and butyrate in the feces of animals with calcium oxalate have changed significantly. Enrichment analysis of these differential metabolites revealed that SCFAs are mainly involved in the metabolic pathways of glyoxylic acid and dicarboxylic acid. This pathway highly overlapped with the metabolic process of the modeling agent ethylene glycol in rats, and ethylene glycol induced stone formation precisely by converting into oxalic acid and its key intermediate products (such as glyoxylic acid and dicarboxylic acid) [36]. In addition, previous studies have reported that after the use of antibiotics causes intestinal flora imbalance and reduces the production of SCFAs, the oxalate deposition in the kidneys of rats significantly increases. Exogenous supplementation of SCFAs can effectively alleviate this phenomenon. The mechanism may be related to up-regulating the expression of the intestinal transporter SLC26A6, enhancing the intestinal oxalic acid excretion capacity, and thereby reducing the oxalic acid load on the kidneys [37]. These results jointly confirm the positive role of SCFAs in the prevention and treatment of calcium oxalate kidney stones. The study demonstrated that PSG mitigated the disruption of intestinal microbiota caused by calcium oxalate, correcting the resultant metabolic dysregulation. This finding provides mechanistic insight into its therapeutic action, which likely involves modulation of the microbiota-metabolite axis.

SCFAs have significant biological activity in kidney diseases. Studies have shown that SCFAs can reduce the generation of reactive oxygen species and the release of inflammatory factors (such as IL-6 and TNF-α) by inhibiting the activity of histone deacetylase and increasing the level of histone acetylation, thereby alleviating renal inflammation and oxidative stress injury and exerting a renal protective effect [38]. To elucidate the protective role of SCFAs against calcium oxalate, this study first employed Pearson correlation analysis to examine the associations between three major SCFAs (acetic, propionic, and butyric acids) and core targets (including *β-arrestin2, NOX4*) identified via transcriptomics, along with other oxidative stress markers. These associations were subsequently validated in *vivo* using an antibiotic-induced gut microbiota depletion model. The results revealed that when microbiota depletion led to insufficient endogenous SCFAs production, the ability of PSG to counteract calcium oxalate-induced oxidative stress was significantly attenuated. Meanwhile, in *vitro* experiments have found that the improvement effect of drug-containing serum prepared under the condition of microbiota depletion on COM induced cell damage is also weaker than that of PSG drug-containing serum prepared under normal intestinal microbiota conditions. The above results suggest that PSG may regulate the occurrence and development of calcium oxalate through the "microbiota—SCFAs—oxidative stress" axis, and its efficacy depends on the complete intestinal microecology.

To identify the bioactive constituents of PSG that enter the systemic circulation and elucidate their functions, this study applied serum pharmacochemistry-based analysis. Traditional research on herbal medicine has largely depended on empirical compound separation and phenotype-based screening [39]. However, the chemical composition of multi-herb formulations is highly complex; moreover, after metabolism by the gut microbiota, numerous bioactive secondary metabolites can be generated, making it difficult to systematically delineate the material basis underlying their efficacy. To address this, the present study integrated serum pharmacochemistry and molecular docking to establish a systematic strategy encompassing identification of serum components and simulation of their potential mechanisms of action. Analysis of serum-PSG subjects identified 170 prototype compounds. Comparison with serum from a microbiota-depleted model (serum-AbP) led to the screening of four key differential constituents. Further mechanistic investigation revealed that emodin activates the *Nrf2* signaling pathway and upregulates antioxidant enzymes such as *HO-1* and *SOD*, thereby scavenging reactive oxygen species and alleviating oxidative damage [40, 41]. Taurodeoxycholic acid has been reported to inhibit stone formation [42], while scutellarin effectively activates *Nrf2* and attenuates oxalate-induced overproduction of ROS [43]. Molecular docking results indicated that all these constituents exhibit favorable binding affinity with *NOX4*, a key oxidative stress-related protein. As a member of the NADPH oxidase family, *NOX4* is widely expressed in the kidney and serves as an important regulator of oxidative stress responses [44]. Building on in *vitro* and in *vivo* validation of PSG efficacy, and in light of existing reports on its circulating components, this study hypothesizes that bioactive metabolites derived from gut microbiota transformation may modulate oxidative stress by directly targeting proteins such as *β-arrestin2*, *MAPK*, and *NOX4*. This mechanism is likely integral to the preventive and therapeutic effects of PSG against calcium oxalate.

The active components derived from PSG after intestinal flora metabolism not only inhibit oxidative stress but also directly modulate the composition of the gut microbiota. Relevant studies have reported that various flavonoid constituents in Baicalensis(such as Scutellarin) also interact closely with the intestinal flora. Flavonoid glycosides undergo deglycosylation by gut microorganisms and are converted into corresponding aglycones. Concurrently, the structure of the gut microbiota undergoes significant changes, characterized by promoting the growth of beneficial bacteria such as *Enterococcus* and *Parabacteroides*, while inhibiting the proliferation of harmful bacteria such as *Shigella*. This underscores the important regulatory role of scutellarin in modulating the microbiota [45, 46]. Emodin, a representative anthraquinone compound, exhibits anti-inflammatory and immunomodulatory activities; however, its oral bioavailability in rats is markedly reduced due to extensive glucuronidation [47]. Since most herbal medicines are administered orally and exert biological effects following interaction with the gut microbiota, emodin likely plays a key role in microbial metabolic processes. In *vitro* single-strain culture experiments have shown that emodin promotes the growth of probiotics such as *Akkermansia, Clostridium, Roseburia, and Ruminococcus*, while exerting inhibitory effects on major intestinal genera such as *Bacteroides* and *Prevotella *[48], indicating its potential in regulating gut microbiota composition. Furthermore, Taurodeoxycholic acid(TUDCA) was detected among the blood-absorbed components. As a bile acid derivative, its presence may be related to the metabolism of herbal constituents by the intestinal flora. Previous studies have demonstrated that TUDCA can ameliorate the reduction in microbial abundance induced by non-alcoholic fatty liver disease by increasing the relative abundance of *Allobaculum, Paraprevotella, Flexispira, and Bifidobacterium*, and by modulating bile acid metabolism [49].In summary, a bidirectional interaction exists between traditional Chinese medicine and the gut microbiota: on one hand, the intestinal flora metabolizes and transforms herbal constituents, generating pharmacologically active substances; on the other hand, these transformed components can in turn feedback-regulate the structure and composition of the gut microbiota, thereby establishing a dynamic and mutually influential relationship.

This study also has certain limitations. Firstly, although the MAPK signaling pathway and its core gene *β-arrestin2* were initially determined through transcriptomic analysis of rat kidneys, the regulatory relationship of upstream and downstream molecules in the β-arrestin2/MAPK/NOX4 signaling pathway was not systematically elucidated. So far, only basic verification has been conducted at the cellular level, that is, after *β-arrestin2* was knocked down, the expression of *p-p38*, *p-ERK, p-JNK* and *NOX4* was observed to be downregulated. Secondly, although the efficacy of PSG was confirmed to be dependent on the complete intestinal microecology through the antibiotic-induced microbiota depletion model, it was not directly demonstrated whether SCFAs mediate the β-arrestin2/MAPK/NOX4 pathway to regulate calcium oxalate-induced oxidative stress injury. Subsequent studies can further supplement the drug administration intervention experiments of SCFAs and their combination with PSG in treating calcium oxalate animals models. Thirdly, in the sequencing analysis of the intestinal microbiota in animal models of calcium oxalate kidney stones, due to the small sample size, only partial changes in the microbiota can be observed, making it difficult to draw universal conclusions. It is necessary to conduct large-scale cohort studies in the future to systematically analyze the characteristics of the gut microbiota in patients with calcium oxalate kidney stones, in order to identify reproducible microbiome markers with clinical predictive value.

## Conclusions

In summary, this study suggests that PSG may alleviate calcium oxalate-induced oxidative stress-induced kidney injury through a multi-target and multi-dimensional regulatory mechanism by acting on the "microbiota—metabolite—oxidative stress" axis. Its potential mechanisms of action mainly include: PSG can restore the composition of intestinal flora, thereby increasing the level of SCFAs, and promoting oxalic acid metabolism by up-regulating the expression of SLC26A6; Meanwhile, the serum active components of PSG transformed by the intestinal flora can inhibit the β-arrestin2/MAPK/NOX4 signaling pathway, thereby suppressing calcium oxalate-induced oxidative stress renal injury (Fig. 11).

## Supplementary Information

Additional file1

## Data Availability

The datasets used in the study are available from the author(Qiushi Cao, email: 2987@hbucm.edu.cn; Bingqi Zhang, email:1,940,332,038@qq.com) on reasonable request.

## Abbreviations

Ab: Antibiotic mixture
AbEN: Calcium oxalate renal stones were formed based on the microbiota depletion model
AbENP: PSG intervened calcium oxalate under conditions of microbiota depletion
BUN: Blood urea nitrogen
Ctrl: Control group
CCK8: Cell counting Kit-8
COM: Calcium oxalate monohydrate
EN: Combining ethylene glycol and ammonium chloride
GO: Gene ontology enrichment analysis
GSEA: Gene set enrichment analysis
H&E: Hematoxylin and eosin
KIM-1: Kidney injury molecule-1
KEGG: Kyoto encyclopedia of genes and genomes enrichment analysis
LDH: Lactate dehydrogenase
MDA: Malondialdehyde
NMDS: Nonmetric multidimensional scaling analysis
Ox: Oxalic acid
Occludin: Intestinal tight junction proteins
OPLSDA: Orthogonal partial least squares discriminant analysis
PSG: Paishi granule
PCA: Principal component analysis
PCoA: Principal co-ordinates analysis
Primary-PSG: PSG herbal stock solution
SHC: Potassium sodium hydrogen citrate
Scr: Serum creatinine
Serum-PSG(Serum-P): PSG-containing serum
Serum-AbPSG(Serum-AbP): PSG-containing serum in microbiota-depleted models
SCFAs: Short-chain fatty acids
SLC26A6: Oxalic acid transport metabolism mediated by member 6 of the oxalic acid transporter solute carrier family 26
T-SOD: Total superoxide dismutase
TCM: Traditional Chinese medicine
UPLC-MS/MS: Ultrahigh-performance liquid chromatography–tandem mass spectrometry
ZO-1: Intestinal tight junction proteins
