# Supplementary material for: Paishi granule inhibits calcium oxalate-induced oxidative stress kidney injury after gut microbiota transformation: a multi-omics analysis combined in vivo and in vitro study
Source: Chin Med. 2026 Jun 25;21:172. doi: 10.1186/s13020-026-01439-4 (PMC13295731; doi:10.1186/s13020-026-01439-4)
Supplement: Supplementary file 1 — Additional file1 [file 13020_2026_1439_MOESM1_ESM.docx]

**Suppelementary Materials and Methods**

1. Analysis process of blood-entering components of PSG

Four experimental groups were established:Blank control serum (Serum-Ctrl); PSG-containing serum(Serum-PSG); PSG-containing serum under microbiota-depleted models (Serum-AbPSG); PSG herbal stock solution (Primary-PSG). Serum-Ctrl group, Serum-PSG group and Serum-AbPSG group were treated with 6 male SD rats of 6 weeks old. After 7 days of adaptive feeding, all animals were respectively treated: Serum-Ctrl group was given normal saline by gavage for 7 days;Serum-PSG group was given 5-fold clinical equivalent dose of PSG by gavage for 7 days;Serum-AbPSG group was given mixed antibiotic solution (ampicillin 0.5 g/L, vancomycin 0.25 g/L, neomycin 0.5 g/L, metronidazole 0.5 g/L) ad libitum to deplete intestinal flora, and then PSG was given by gavage for 7 days. After treatment, serum samples from each group were collected by centrifugation at 3000 rpm for 15 minutes at 4℃.

Metabolite detection in serum was performed using ultra-high performance liquid chromatography (UPLC-MS/MS) technology. This project uses the Vanquish (Thermo Fisher Scientific) ultra-high performance liquid chromatograph. The target compounds are separated by chromatography through a Phenomenex Kinetex C18 (2.1 mm×100 mm, 2.6μm) liquid chromatography column. The A phase of the liquid chromatography is an aqueous solution containing 0.01% acetic acid, and the B phase is isopropanol: acetonitrile (1:1, v/v). The sample plate temperature is 4 ℃, and the injection volume is 2μL. Network pharmacology analysis and Molecular docking were performed in conjunction with public databases to identify potential targets of action for blood components.

1. Network pharmacology analysis and Molecular dock

Based on the analysis of traditional Chinese medicine (TCM)-derived serum components, the identified serum metabolites were used to predict potential targets by querying the TCMSP (https://old.tcmsp-e.com/tcmsp.), CHEMBL (https://www.ebi.ac.uk/CHEMBL/), and PubChem (https://pubchem.ncbi.nlm.nih.gov/) databases. Concurrently, disease targets associated with calcium oxalate kidney stones were retrieved from the GeneCards (https://www.genecards.org/), OMIM (https://www.omim.org/), and TTD (https://idrblab.net/ttd/) databases. The intersection of drug-predicted targets and disease-related targets was identified as common targets and imported into the STRING database for protein–protein interaction (PPI) analysis. The top 100 core genes ranked by Degree value were selected to construct a PPI network. These genes were subsequently submitted to the Gene Ontology (GO) and KEGG Pathway databases for functional and pathway enrichment analysis. A multi-dimensional “drug–component–target–pathway” network pharmacology model was built using Cytoscape, and the top 10 key active components were identified based on target enrichment counts.

Using the resulting network, components with high Degree values were designated as core targets. Three-dimensional structures of the corresponding proteins were downloaded from the Protein Data Bank (PDB), and ligand structures of the active components were obtained from PubChem. Molecular docking was performed using AutoDock Tools 1.5.7, where receptor proteins and small-molecule ligands were prepared by adding hydrogen atoms and removing water molecules. Docking parameters were configured to ensure comprehensive coverage of the protein binding site. The optimal ligand–receptor binding conformation, as determined by binding energy, was visualized and analyzed using Chimera 1.17.3.

1. Fecal genomic DNA testing

（1）Transfer 200 mg of fecal sample into a 1.5mL centrifuge tube.

（2）Perform DNA extraction according to the instructions of the "Fecal Genome DNA Extraction Kit."（TIANamp Stool DNA Kit; NO:DP328-02）

（3）The final DNA elution volume is 50 μL.

（4）Take 5 μL of the extracted DNA for agarose gel electrophoresis using a 1% agarose gel, and run at a constant voltage of 135 V for 30 minutes (DNA marker: DL2000).

（5）Place the gel in a imaging system for photography.

1. siRNA transfection experiment

In this study, Lipofectamine™ 2000 transfection reagent was used to deliver targeted siRNA fragments into rat renal tubular epithelial cells (NRK-52E). Cells were seeded in 6‑well plates at a density of 2 mL per well and maintained in a 37 °C, 5% CO₂ incubator under standard culture conditions. Prior to transfection, the original medium was removed and replaced with serum‑free DMEM. The transfection mixtures were prepared as follows: 10µL of siRNA stock solution (20µM) was diluted in 100µL of serum‑free Opti‑MEM® I Medium and mixed gently. Separately, 5µL of Lipofectamine™ 2000 reagent was diluted in 100µL of serum‑free Opti‑MEM® I Medium and incubated at room temperature for 5 minutes. The two dilutions were then combined in equal volumes, mixed thoroughly to form a 200µL transfection complex, and added dropwise to each well. Cells were returned to the 37 °C, 5% CO₂ incubator for continued incubation. Following the transfection period, total cellular protein was extracted for subsequent Western blot analysis.The sequence of the small fragments is shown in Table 7.

1. Effect of PSG on intestinal microflora in rats with calcium oxalate kidney stone and Lefse analysis

Lefse is a algorithm used for discovering high-dimensional biological markers and revealing genomic characteristics. Including genes, metabolism and classification, it is used to distinguish two or more biological conditions (or groups). This algorithm emphasizes statistical significance and biological correlation. Enable researchers to identify features of different abundances and the associated categories.

Lefse endows it with powerful recognition capabilities through biological statistical differences. Then, it conducts additional tests to assess whether these differences are in line with the expected biological behavior. Specifically, the non-parametric factorial Kruskal-Wallis (KW) sum-rank test (non-parametric factorial Kruskal-Wallis and rank test) is first used to detect the characteristics with significant abundance differences and identify the groups with significant differences from the abundance. Finally, Lefse employed linear discriminant analysis (LDA) to estimate the extent to which the abundance of each component (species) affected the differential effect.

The statistical results of Lefse consist of two parts: the bar chart of LDA value distribution, the evolutionary branch chart (phylogenetic distribution), and the comparison of the abundance of biomarkers with statistical differences between groups in different groups.


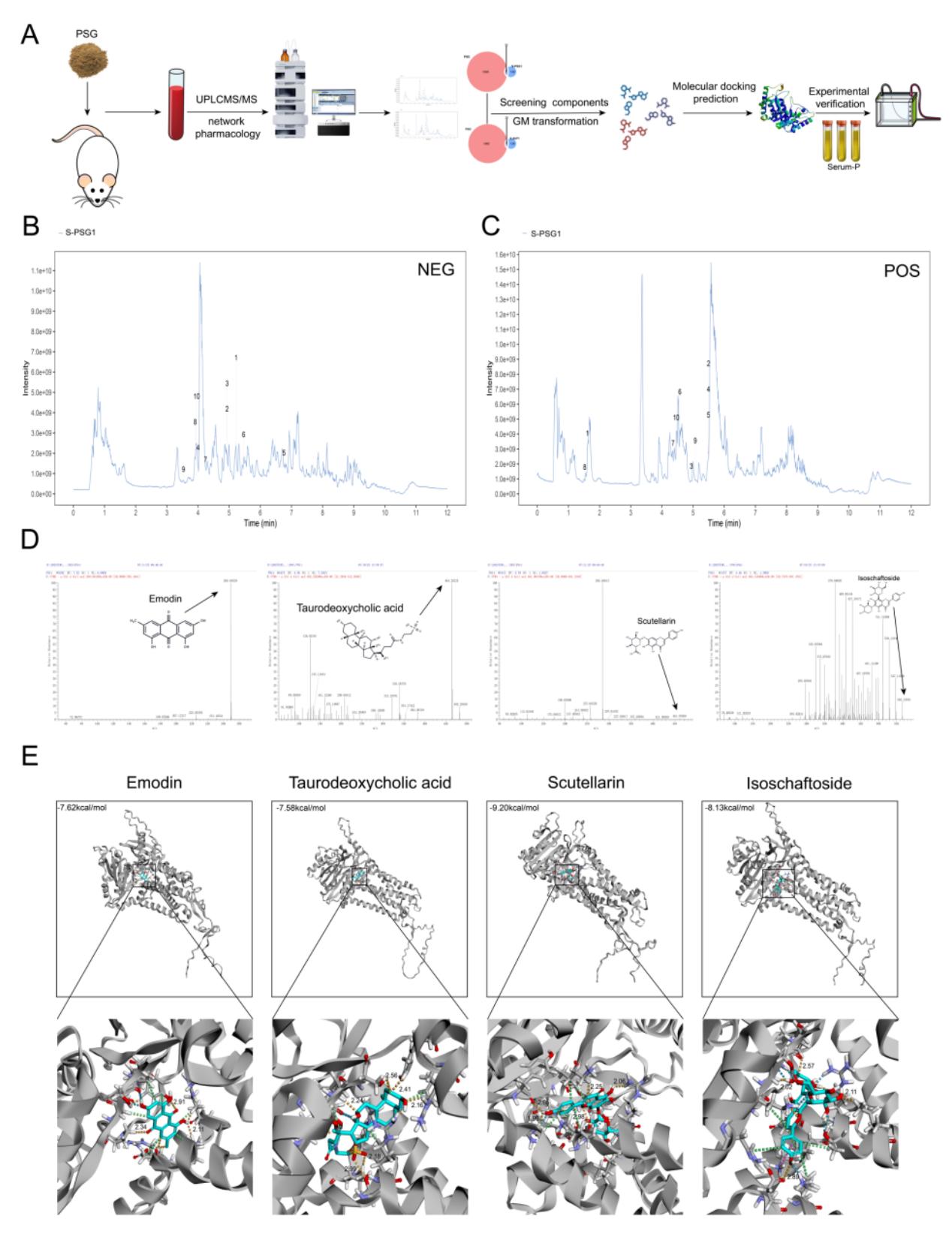


Figure. S1. Integrated UPLC-MS/MS and network pharmacology identified the key therapeutic compounds in serum PSG. (A) Schematic diagram of the experimental workflow. (B) TIC chromatogram of serum-PSG sample1 in negative ion mode(NEG).（C）TIC chromatogram of serum-PSG sample1 in positive ion mode(POS). (D) Four differential components were screened between PSG serum and AbPSG serum. (E) Molecular docking models of NOX4 with Emodin、Taurodeoxycholic acid、Scutellarin and Isoschaftoside.

**S-Table3: Analysis of main components in serum-PSG by UPLC-MS/MS**

| **Name** | **MZMED（m/z）** | **RTMED（S）** | **Type** | **Number** | **Formula** |
| --- | --- | --- | --- | --- | --- |
| *Brassicin* | 955.213609027752 | 313.2915 | N-TIC | 1 | C22H22O12 |
| *Citreorosein* | 285.03965488984 | 296.063 | N-TIC | 2 | C15H10O6 |
| *Flavonol base + 5O, O-Hex* | 479.082271290978 | 295.958 | N-TIC | 3 | C21H20O13 |
| *2-hydroxy-3-methoxy-benzaldehyde* | 188.997298167106 | 240.359 | N-TIC | 4 | C8H8O3 |
| *Emodin* | 269.04519683863 | 405.6945 | N-TIC | 5 | C15H10O5 |
| *Scutellarin* | 483.053331245084 | 327.9825 | N-TIC | 6 | C21H18O12 |
| *Homogentisic acid* | 187.060654421254 | 254.539 | N-TIC | 7 | C8H8O4 |
| *Methylgallate* | 367.06629493675 | 234.473 | N-TIC | 8 | C8H8O5 |
| *(2R,3S,4S,5R,6R)-2-(hydroxymethyl)-6-[[(2R,3S,4S,5R,6S)-3,4,5-trihydroxy-6-(3-hydroxy-5-methyl-phenoxy)tetrahydropyran-2-yl]methoxy]tetrahydropyran-3,4,5-triol* | 464.177359819272 | 212.063 | N-TIC | 9 | C19H28O12 |
| *Acaciabiuronic acid* | 355.091510392659 | 237.347 | N-TIC | 10 | C12H20O12 |
| *Hordenine* | 166.122400462772 | 96.4521 | P-TIC | 1 | C10H15NO |
| *Isoquercetrin* | 465.102493266821 | 329.763 | P-TIC | 2 | C21H20O12 |
| *5,7-dihydroxy-2-(4-hydroxyphenyl)-3-[(2S,3R,4S,5S)-3,4,5-trihydroxytetrahydropyran-2-yl]oxy-chromen-4-one* | 465.102698179504 | 296.63 | P-TIC | 3 | C20H18O10 |
| *1,2-Methylenedioxy-3,4,6-Trimethoxydibenzofuran* | 303.085783428405 | 329.709 | P-TIC | 4 | C16H14O6 |
| *(±)-Naringenin* | 290.099548040848 | 329.5545 | P-TIC | 5 | C15H12O5 |
| *Methyl jasmonate* | 225.148308293482 | 274.54 | P-TIC | 6 | C13H20O3 |
| *Taurodeoxycholic acid* | 464.28232 | 261.434 | P-TIC | 7 | C26H45NO6S |
| *Galactose* | 181.070268761624 | 91.3449 | P-TIC | 8 | C6H12O6 |
| *Riboflavin* | 359.133043096549 | 304.199 | P-TIC | 9 | C17H20N4O6 |
| *Isoschaftoside* | 565.154166827715 | 267.396 | P-TIC | 10 | C26H28O14 |


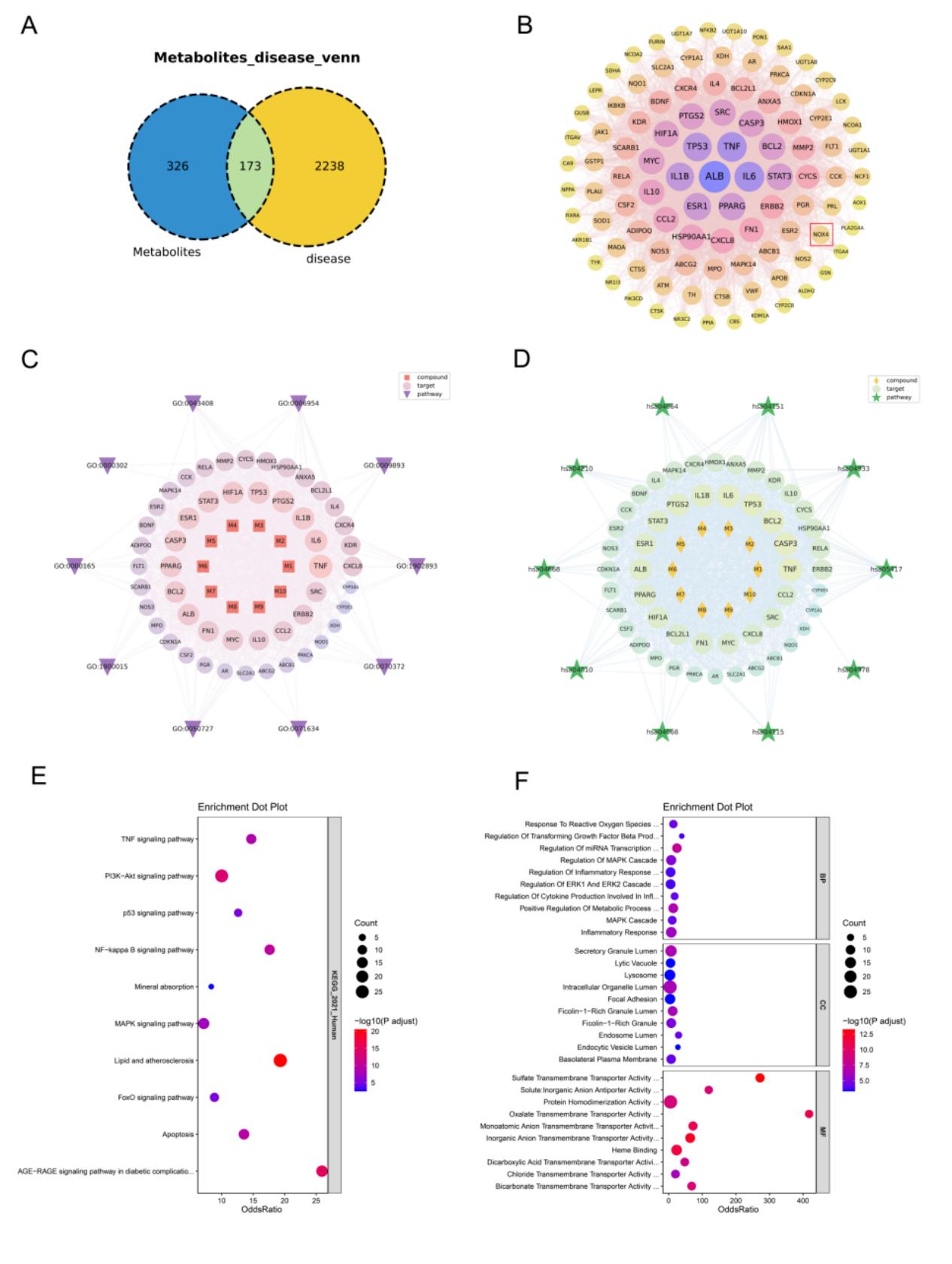


Figure. S2. Network pharmacology analysis of serum-PSG. (A) Metabolite target and disease target venn plot. (B) PPI protein intermapping. (C) Network-target-GO enrichment pathway map. (D) Network-target-KEGG enrichment pathway map. (E) KEGG enrichment analysis. (F) GO enrichment analysis

**S-Table4: Top 10 substances in the number of gene targets enriched by core components of PSG**

| **Compound id** | **Name** | ***gene*_count** |
| --- | --- | --- |
| M1 | *Emodin* | 26 |
| M2 | *Scutellarin* | 16 |
| M3 | *(±)-Naringenin* | 11 |
| M4 | *Citreorosein* | 5 |
| M5 | *Taurodeoxycholic acid* | 3 |
| M6 | *Methyl jasmonate* | 3 |
| M7 | *Methylgallate* | 3 |
| M8 | *Homogentisic acid* | 2 |
| M9 | *Riboflavin* | 2 |
| M10 | *Isoschaftoside* | 2 |

**S-Table5: GO enrichment analysis BP entries**

| **ID** | **Name** | **P.adjust** |
| --- | --- | --- |
| GO:0000302 | Response To Reactive Oxygen Species | 0.0000511616204126372 |
| GO:0071634 | Regulation Of Transforming Growth Factor Beta Production | 0.000179073286849126 |
| GO:1902893 | Regulation Of miRNA Transcription | 1.96961071221721E-08 |
| GO:0043408 | Regulation Of MAPK Cascade | 0.0000104660042042089 |
| GO:0050727 | Regulation Of Inflammatory Response | 0.000170101717000416 |
| GO:0070372 | Regulation Of ERK1 And ERK2 Cascade | 0.00018448301357253 |
| GO:1900015 | Regulation Of Cytokine Production Involved In Inflammatory Response | 0.0000741995857972011 |
| GO:0009893 | Positive Regulation Of Metabolic Process | 3.33941195590655E-07 |
| GO:0006954 | Inflammatory Response | 1.36527487666461E-06 |
| GO:0000165 | MAPK Cascade | 0.0000626231334037692 |

**S-Table6: KEGG enrichment analysis entries**

| **ID** | **Name** | **P.adjust** |
| --- | --- | --- |
| hsa04668 | TNF signaling pathway | 2.26255091914736E-09 |
| hsa04151 | PI3K-Akt signaling pathway | 1.93588246008178E-14 |
| hsa04115 | p53 signaling pathway | 0.0000102458584616041 |
| hsa04064 | NF-kappa B signaling pathway | 9.20744885907398E-11 |
| hsa04978 | Mineral absorption | 0.0034461588810013 |
| hsa04010 | MAPK signaling pathway | 3.35752746246614E-08 |
| hsa05417 | Lipid and atherosclerosis | 2.6037688542002E-21 |
| hsa04068 | FoxO signaling pathway | 6.92920092517447E-06 |
| hsa04210 | Apoptosis | 2.83402911819508E-10 |
| hsa04933 | AGE-RAGE signaling pathway in diabetic complications | 5.98025001863386E-16 |

**S-Table7：The sequence of the siRNA**

| **Gene** | **Sense** | **Antisense** |
| --- | --- | --- |
| SiRNA-NC | UUCUCCGAACGUGUCACGUTT | ACGUGACACGUUCGGAGAATT |
| Si-β-arrestin2（1） | GAGAAGACCUGGAUGUACUTT | AGUACAUCCAGGUCUUCUCTT |
| Si-β-arrestin2（2） | GUGCGGCUUAUCAUCAGAATT | UUCUGAUGAUAAGCCGCACTT |
| Si-β-arrestin2（3） | GCUUCCAGCACCAUUGUGATT | UCACAAUGGUGCUGGAAGCTT |
| Si-β-arrestin2（4） | GAAUUCGAUACCAACUAUGTT | CAUAGUUGGUAUCGAAUUCTT |


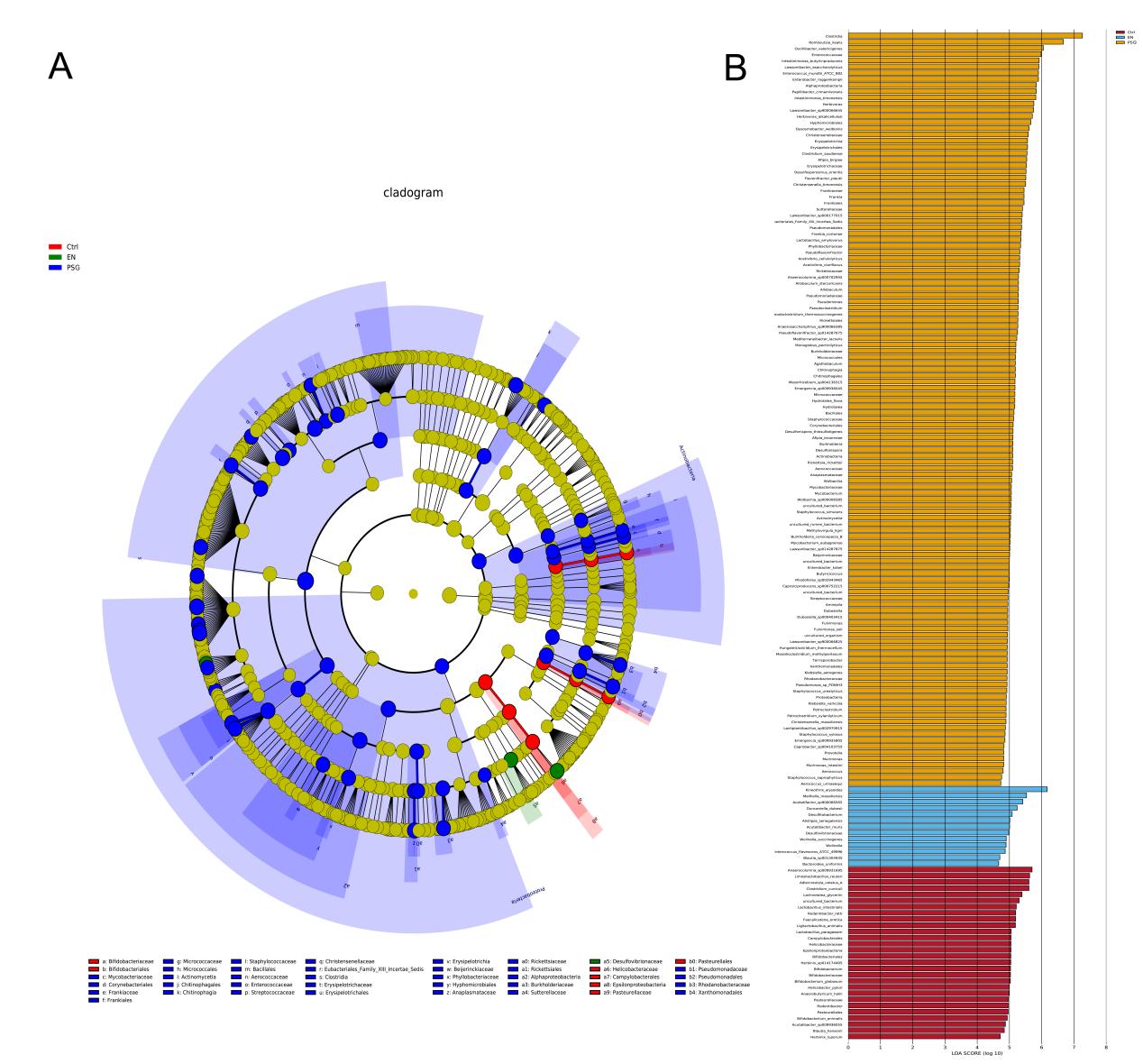


Figure. S3. Abundance analysis of significantly enriched groups by linear discriminant analysis effect values (Lefse) and linear discriminant analysis (LDA). (A)Lefse was used to identify the characteristic flora among experimental groups, and the circle size was proportional to the relative abundance of each flora. (B)The LDA method with threshold scores greater than 3 was used to show significant changes in intestinal flora abundance in each group. The column length in the LDA plot represents the influence degree of significantly different species among groups.


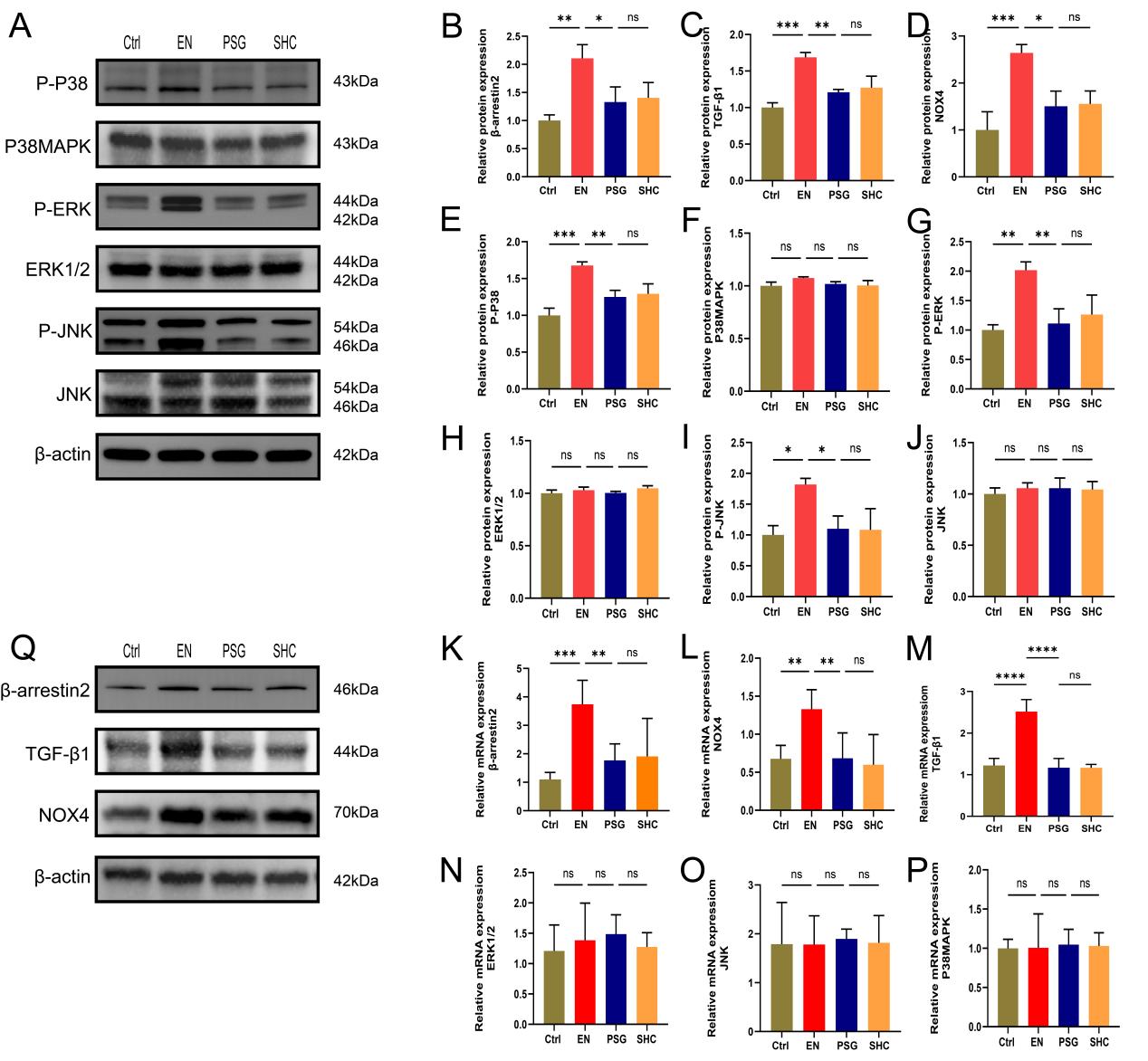


Figure. S4. Effect of Paishi granule (PSG) on oxidative damage induced by calcium oxalate kidney stones. (A) Effect of Western blott band reaction PSG on MAPK kinase in rat kidney stones. (B-D) Quantitative analysis of relative expression of *β-arrestin2, TGF-β1* and *NOX4* proteins. (E-J) Quantitative analysis of relative expression of proteins associated with MAPK signaling pathway. (K-P) PCR analysis of *β-arrestin2, TGF-β1, NOX4 and JNK, ERK1/2*, and *P38MAPK* genes. (Q) Western blott band response expression of *β-arrestin2, TGF-β1* and *NOX4* proteins. Data are expressed as mean ± standard deviation (n=6).**P*<0.05, ***P*<0.01, ****P*<0.001 compared with EN, and ^ns^*P*>0.05 compared with PSG.


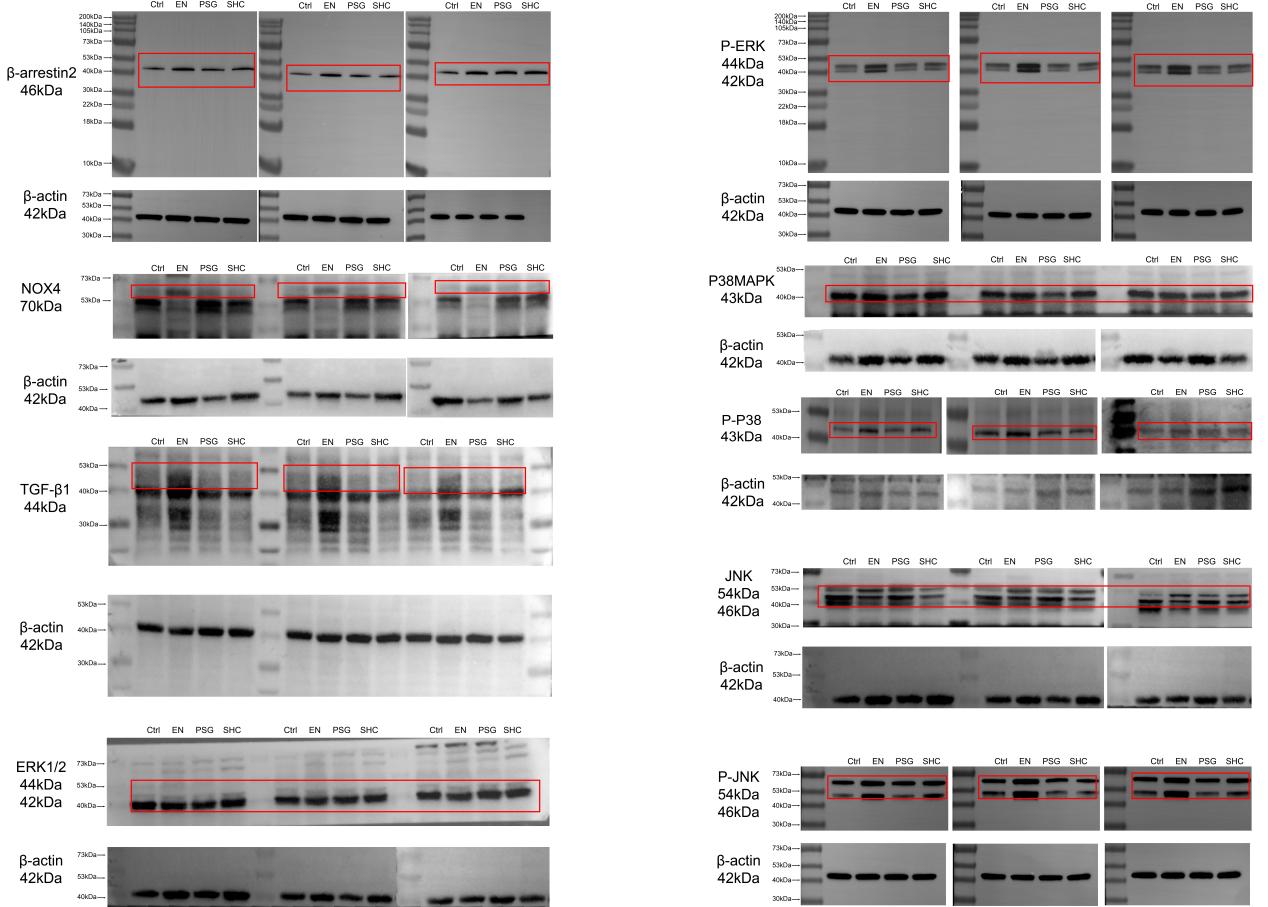


Fig. S5. Western blot of kidney tissue. Western blot results of *NOX4* (70kDa), *β-arrestin2* (46 kDa), *TGF-β1* (44kDa), *phosphorylated-ERK* (42, 44kDa), *ERK1/2* (42, 44kDa), *phosphorylated- P38* (43kDa), *P38MAPK* (43kDa), *phosphorylated-JNK* (46, 54kDa) and *JNK* (46, 54kDa) in kidney tissues of rats in four experimental groups (Ctrl group, EN group, PSG group and SHC group).


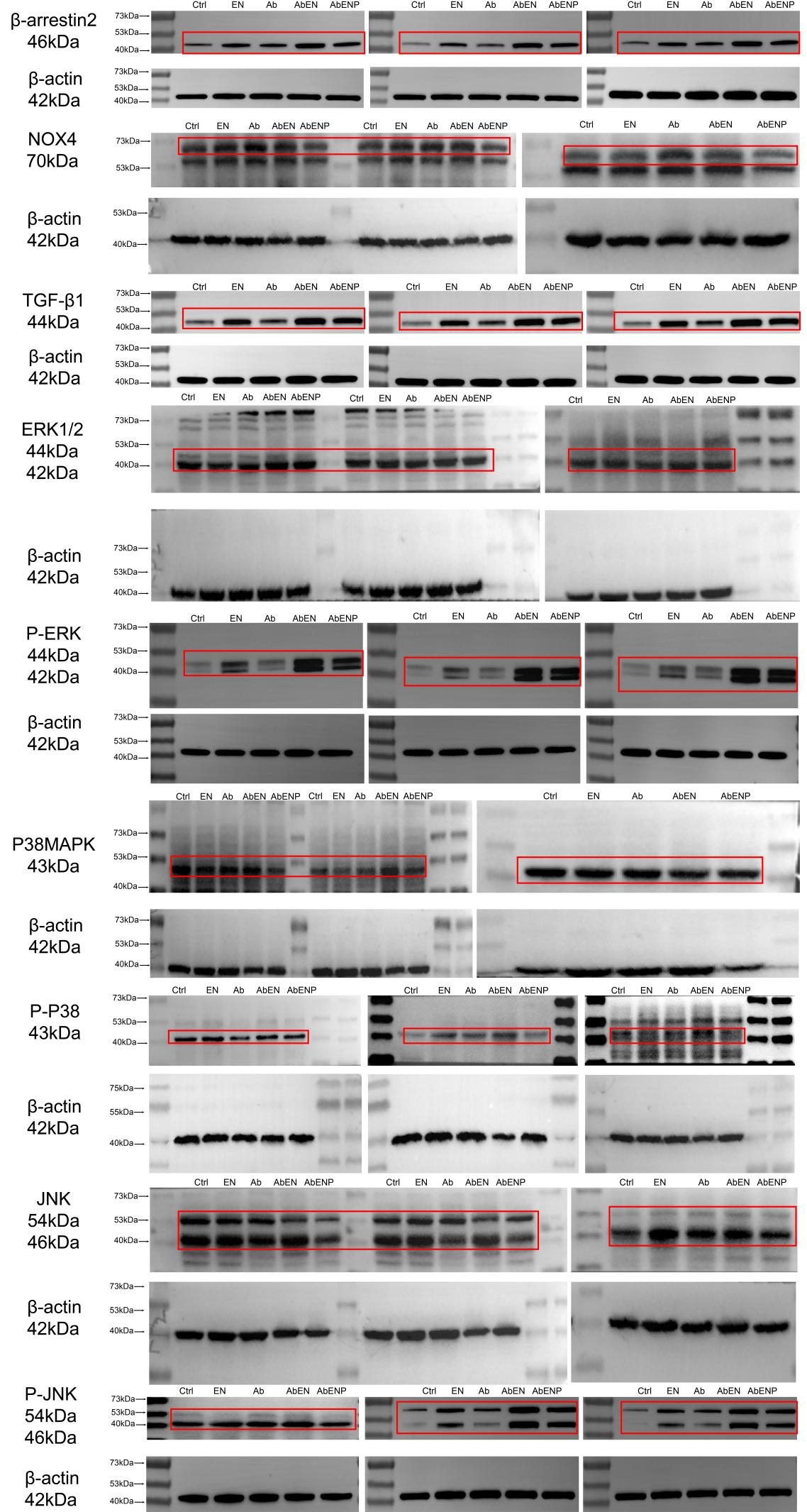


Fig. S6. Western blot of kidney tissue. Western blot results of *NOX4* (70 kDa), *β-arrestin2* (46 kDa), *TGF-β1* (44kDa), *phosphorylated-ERK* in rat kidney tissue (42, 44 kDa), *ERK1/2* (42, 44 kDa), *phosphorylated-P38* (43 kDa), *P38MAPK* (43 kDa), *phosphorylated-JNK* (46, 54 kDa), *JNK* (46, 54 kDa)in kidney tissues of rats in five experimental groups(Ctrl group, EN group, Ab group, AbEN group, AbENP group ).

.
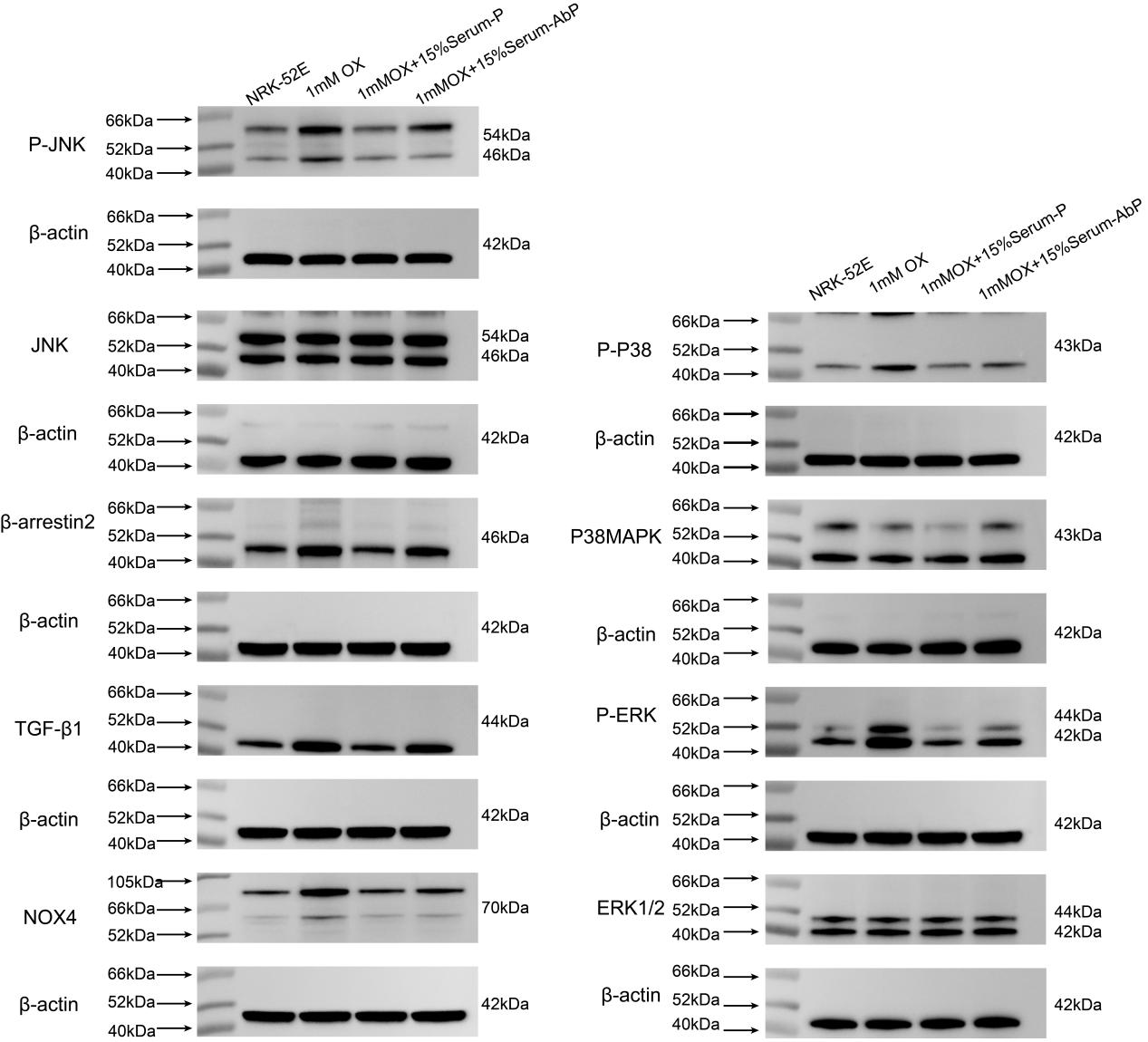


Fig. S7. Western blot of NRK-52E. Western blot results of *NOX4* (70 kDa), *β-arrestin2* (46 kDa), *TGF-β1* (44kDa), *phosphorylated-ERK* (42, 44kDa), *ERK1/2* (42, 44kDa), *phosphorylated-P38* (43kDa), *P38MAPK* (43 kDa), *phosphorylated-JNK* (46, 54kDa), *JNK* (46, 54kDa)in NRK-52E of four experimental groups(NRK-52E group, 1mMOx group, 1mMOx+15%Serum-P group, 1mMOx+15%Serum-AbP group ).


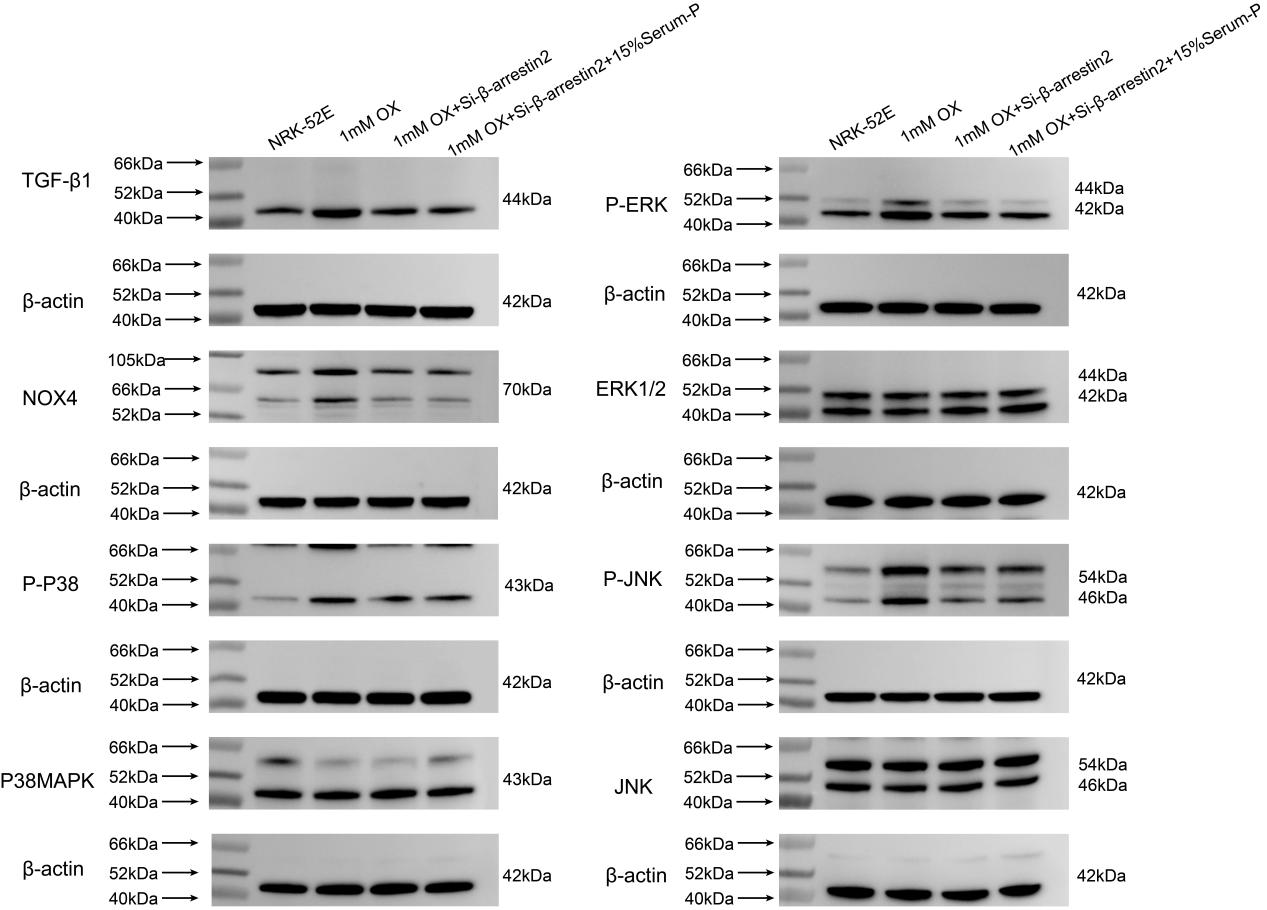


Fig. S8. Western blot of NRK-52E. Western blot results of *NOX4* (70 kDa), *TGF-β1* (44kDa), *phosphorylated-ERK* (42, 44kDa), *ERK1/2* (42, 44 kDa), *phosphorylated-P38* (43kDa), *P38MAPK* (43 kDa), *phosphorylated-JNK* (46, 54kDa), *JNK* (46, 54kDa)in NRK-52E of

four experimental groups(NRK-52E group, 1mMOx group, 1mMOx+Si-β-arrestin2 group, 1mMOx+Si-β-arrestin2+15%Serum-P group ).


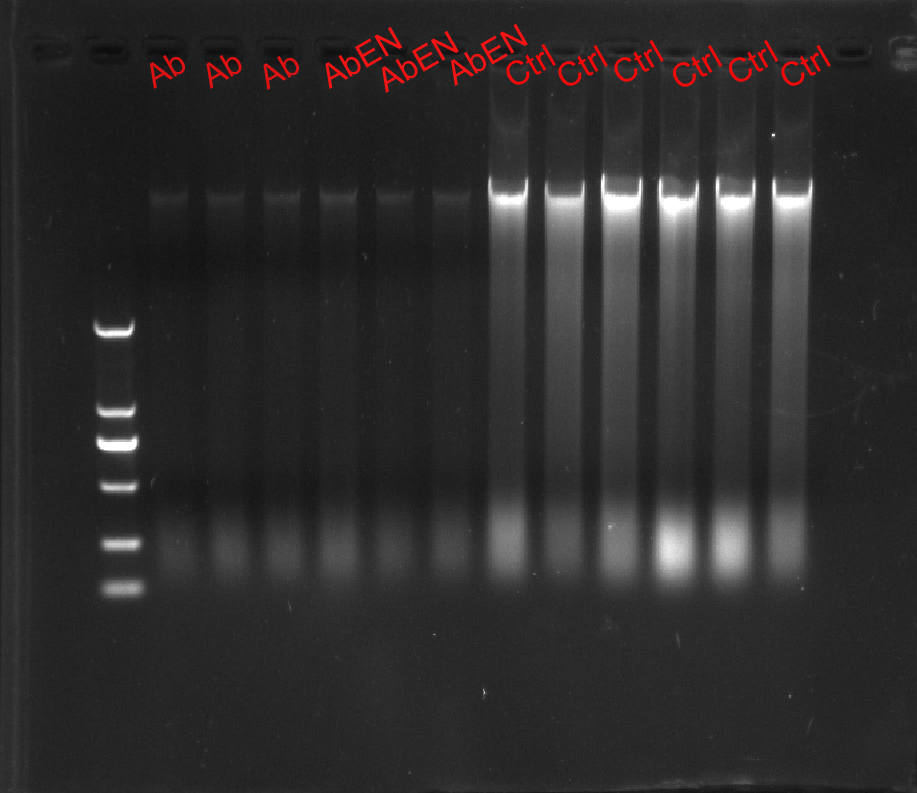


Fig. S9. Detection of fecal genomes in animal models of antibiotic-induced microbiota depletion. The fecal gene content in the normal group（Ctrl） was significantly higher than that in the group treated with antibiotics（Ab or AbEN). The research results suggest that the richness of the microbiota in the feces of rats treated with antibiotics decreased significantly.


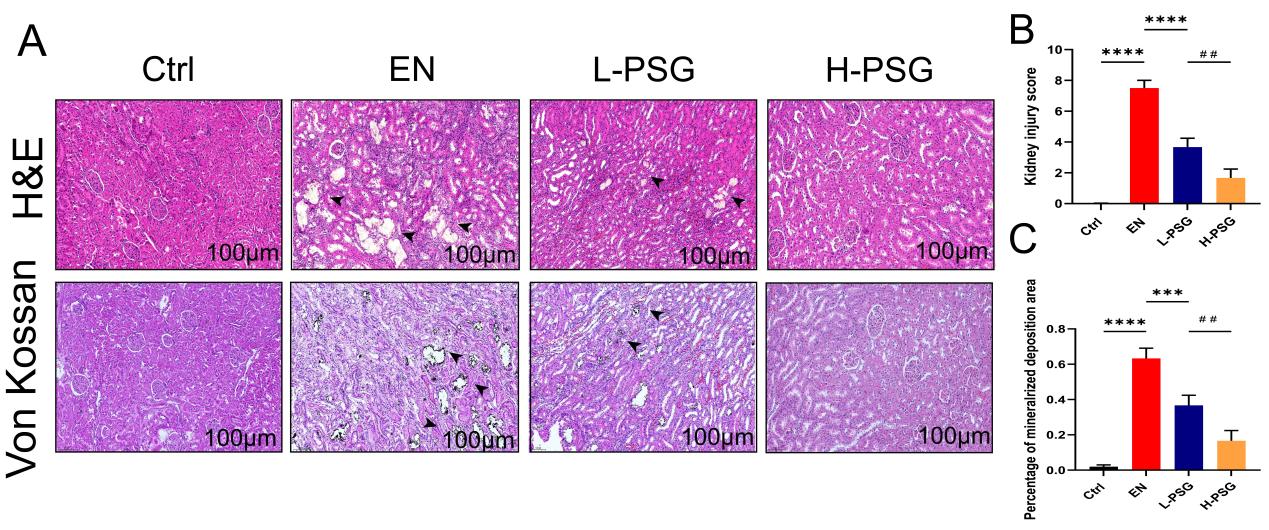


Fig. S10. Design a pre-experiment to screen the dose gradient concentration of PSG efficacy.（A）H&E and Von Kossan staining of rat kidneys(scale 100 µm, 200×).（B）Kidney injury score.（C）Mineralized deposition area. Data are presented as mean ± standard deviation (n=3). **P*<0.05, ***P*<0.01, ****P*<0.001 compared with EN, ^##^*P*＜0.01 compared with H-PSG. The representative concentrations of H-PSG and L-PSG are 1.53g/mL and 0.75g/mL respectively.


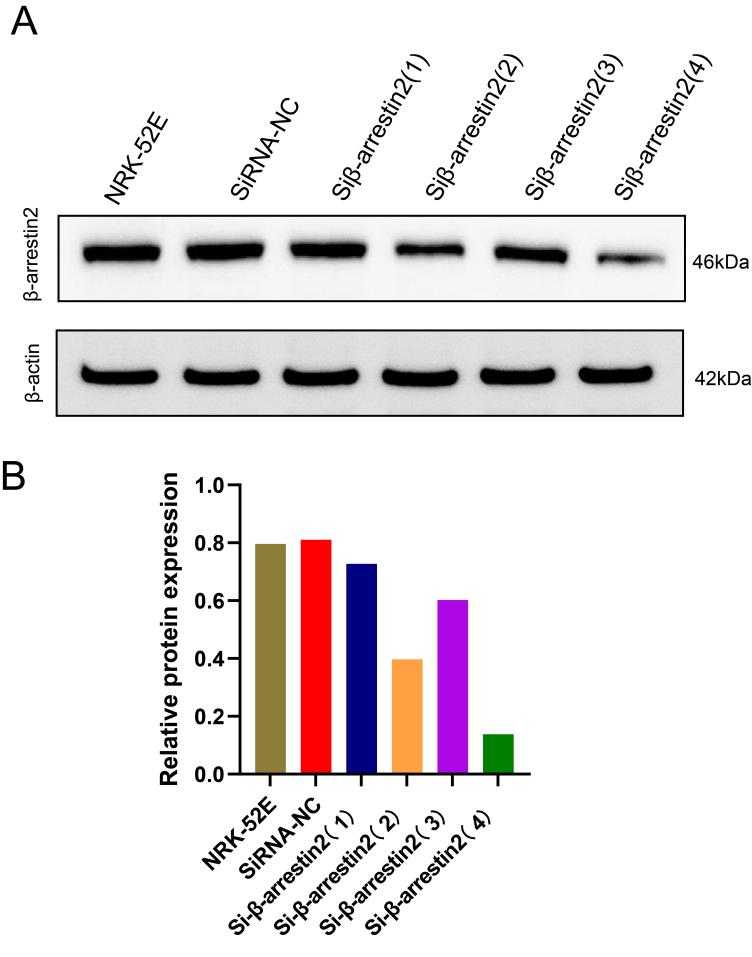


Fig. S11. siRNA gene knockdown verification experiment.（A）The Western blot experiment was used to verify the expression of *β-arrestin2* protein. （B）Quantitative analysis of *β-arrestin2* protein.
